# Specific recognition mechanism of an antibody to sulfated tyrosine and its potential use in biological research

**DOI:** 10.1016/j.jbc.2025.108176

**Published:** 2025-01-10

**Authors:** Kan Ujiie, Makoto Nakakido, Seisho Kinoshita, Jose M.M. Caaveiro, C. Kevin Entzminger, Shigeru C.J. Okumura, Toshiaki Maruyama, Kosuke Miyauchi, Tetsuro Matano, Kouhei Tsumoto

**Affiliations:** 1 Department of Bioengineering, School of Engineering, The University of Tokyo, Tokyo, Japan; 2 Department of Protein Drug Discovery, Graduate School of Pharmaceutical Sciences, Kyushu University, Fukuoka, Japan; 3 Abwiz Bio, Inc, San Diego, California, USA; 4 AIDS Research Center, NIID, Tokyo, Japan; 5 Institute of Medical Science, The University of Tokyo, Tokyo, Japan; 6 Department of Chemistry and Biotechnology, School of Engineering, The University of Tokyo, Tokyo, Japan; 7 Medical Device Development and Regulation Research Center, School of Engineering, The University of Tokyo, Tokyo, Japan

**Keywords:** post-translational modification, sulfated tyrosine, antibody, physicochemical analyses, crystal structure, molecular dynamics simulation, HIV infection

## Abstract

Post-translational modification of proteins is a crucial biological reaction that regulates protein functions by altering molecular properties. The specific detection of such modifications in proteins has made significant contributions to molecular biology research and holds potential for future drug development applications. In HIV research, for example, tyrosine sulfation at the N-terminus of C-C chemokine receptor type 5 (CCR5) is considered to significantly enhance HIV infection efficiency. However, antibodies specific to sulfated CCR5 still need to be developed. In this study, we successfully generated an antibody that specifically recognized the sulfated N-terminal peptide of CCR5 through rabbit immunization and panning *via* phage display using a CCR5 N-terminal peptide containing sulfate modification. We used various physicochemical methods in combination with molecular dynamics simulation to screen for residues that could be involved in recognition of the sulfated peptide by this antibody. We also confirmed that this antibody recognized the sulfated full-length CCR5 on the cell surface, which suggested it should be useful as a research tool that could lead to the development of novel therapeutics. Although the antibody binding did not inhibit HIV infection, it could be also described as sulfation site-specific binding, beyond sulfation-specific binding.

Proteins undergo various post-translational modifications (1) such as phosphorylation (2), acetylation (3), methylation (4), and glycosylation (5, 6). These modifications play critical roles in regulating protein functions by modulating protein stability, localization, and molecular interactions. One such post-translational modification is the sulfation of tyrosine residues, which is crucial in a variety of biological responses, including signal transduction, immune response, and viral infection (7, 8). Because the sulfate group is highly acidic and exhibits strong electron-withdrawing properties, tyrosine sulfation generates additional intramolecular and intermolecular interactions with various proteins, leading to the regulation of biological processes (9).

Tyrosine sulfation is mediated by protein tyrosine sulfotransferases (TPSTs) in the Golgi apparatus, where the active sulfate donor promotes the transfer of the sulfate group (10, 11, 12). The two isoforms of TPST (named TPST1 and TPST2) share nearly 60% sequence homology (13, 14). Although structural analysis revealed that the overall structure and the amino acid residues in enzyme active sites responsible for tyrosine recognition and sulfation are nearly identical between the two isoforms, Tanaka *et al.* (14) found that the *in vitro K*_m_ value for TPST2 activity was slightly higher than that of TPST1. Given that TPST1 and TPST2 exhibit different expression levels across tissues and have distinct optimal reaction conditions, such as pH level, for their sulfation activity on the same peptide ligands, they may have complementary roles in different tissues and environmental contexts within the body (15). However, the molecular mechanism responsible for the regulation of sulfation remains to be elucidated.

Tyrosine sulfation has a significant impact on protein function *in vivo*, and the sulfation of the G protein-coupled C-C chemokine receptor type 5 (CCR5) has been particularly well-studied (16, 17, 18, 19). CCR5 is expressed on the surface of T cells and functions as a β-chemokine receptor, playing a crucial role in regulating the immune system. CCR5 is also a target for viral infections, including HIV (17). The initial step of HIV infection involves the binding of the HIV glycoprotein gp120 to both the N-terminal regions of CCR5 and CD4 (19). The anti-HIV drug Maraviroc inhibits HIV infection by binding to CCR5 and blocking its interaction with gp120 (20, 21). The N-terminal region of CCR5 contains four tyrosine residues, and their sulfation has been shown to increase HIV infection efficiency by approximately 50-fold (17). Infection experiments using T cells expressing CCR5 mutants in which tyrosine residues are replaced with phenylalanine or aspartic acid to mimic non-sulfated CCR5 suggest that each tyrosine sulfation contributes to HIV entry (17). Furthermore, previous studies showing the complex structure of gp120 and CCR5 indicate that sulfate groups play roles in the interaction between the N-terminal region of CCR5 and gp120 (16). However, antibodies capable of detecting sulfated tyrosine residues in a sequence-specific manner had not been developed before this study, leaving many aspects of the sulfation status of CCR5 on cells unclear. Additionally, antibodies that specifically recognize the sulfated N-terminal region of CCR5 could be applied to assessing HIV susceptibility and developing novel HIV entry inhibitors.

In this study, we successfully generated an antibody that specifically recognizes the sulfated CCR5 through rabbit immunization and panning *via* phage display using a sulfated tyrosine-containing CCR5 N-terminal peptide. We elucidated the recognition mechanism of this antibody using various biophysical methods, such as surface plasmon resonance (SPR), isothermal titration calorimetry (ITC), structural analyses, and molecular dynamics (MD) simulation and identified the main sulfate group recognized by the antibody. We also confirmed that this antibody can specifically recognize sulfation modifications on CCR5 expressed on the cell surface and examined its inhibitory effects on viral infection.

## Results

### Generation of sulfated tyrosine-specific antibodies

**Figure 1:**
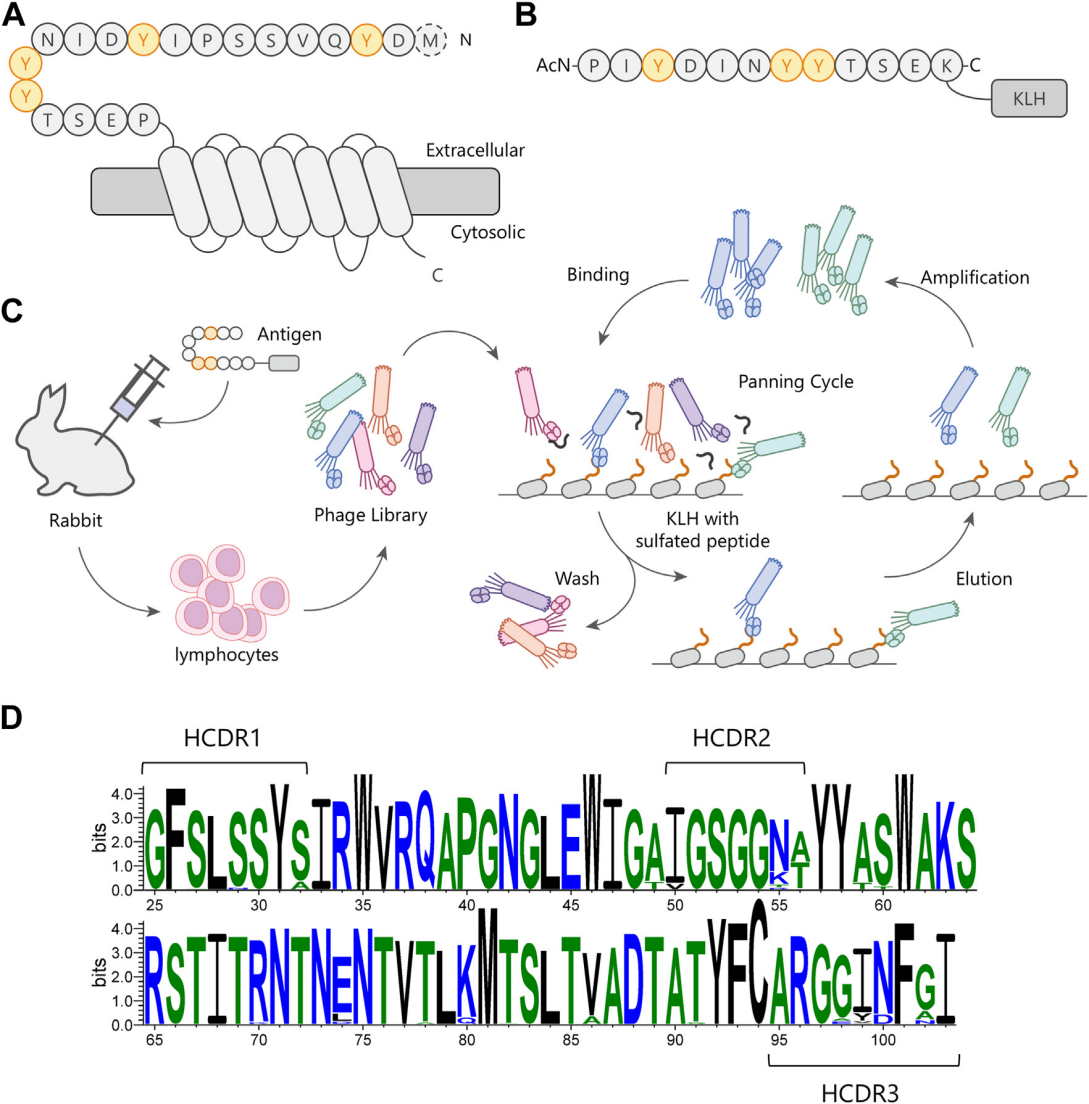
**Acquisition of sulfation-specific antibodies using rabbit immunization followed by phage display-based selection.***A*, schematic diagram of the N-terminal sequence of CCR5. There are four Tyr residues, each of which can potentially be sulfated. *B*, antigen used for immunization. The N-terminal peptide of CCR5 was conjugated to the N-terminus of KLH. *C*, schematic diagram of phage display. The DNA library of antibodies was constructed by extracted RNA from lymphocytes of immunized rabbit. Phages that displayed candidate antibodies were selected by immobilized antigens. *D*, clustering of antibody sequences obtained through phage display. The sequences from CDR1 to CDR3 were highly convergent.

Fig. 1*A* shows the N-terminal sequence of CCR5. To obtain antibodies that specifically recognize the sulfated tyrosines on the N-terminal sequence of CCR5, we synthesized a peptide containing Pro8 to Ser18 with sulfated Tyr10, Tyr14, and Tyr15 to be used as an immunogen (Fig. 1*B*). Based on the synthesized peptide sequence, we renumbered the amino acid 8Pro as 1Pro and 18Ser as 11Ser.

We raised antibodies using the synthesized sulfated N-terminal peptide of CCR5 as previously described (22). Briefly, the peptide was conjugated with keyhole limpet hemocyanin (KLH), an adjuvant antigen, and rabbits were immunized with the KLH-peptide conjugate (22). After the serum titer levels were elevated, we extracted RNA to create an immune library by excising the fragment antigen-binding region (Fab) fragment antibody portions. We then conducted panning *via* phage display using the antigen employed for immunization (Fig. 1*C*) and also cells overexpressing CCR5 and TPSTs (23). We evaluated the binding of the antibodies using ELISA and flow cytometry and selected antibody sequences that bound to both the immunogen peptide and sulfated CCR5 expressed on the cell surface. Sequence alignment of the selected antibody sequences revealed that all sequences were nearly identical, resulting in the successful identification of a single antibody as a hit clone (Fig. 1*D*, Fig. S1) (24). From this converged sequence, we chose a representative clone (BA8) as the sulfated tyrosine-specific antibody for further analysis.

### The binding specificity of the BA8 clone for the sulfated *versus* the non-sulfated peptide

**Figure 2:**
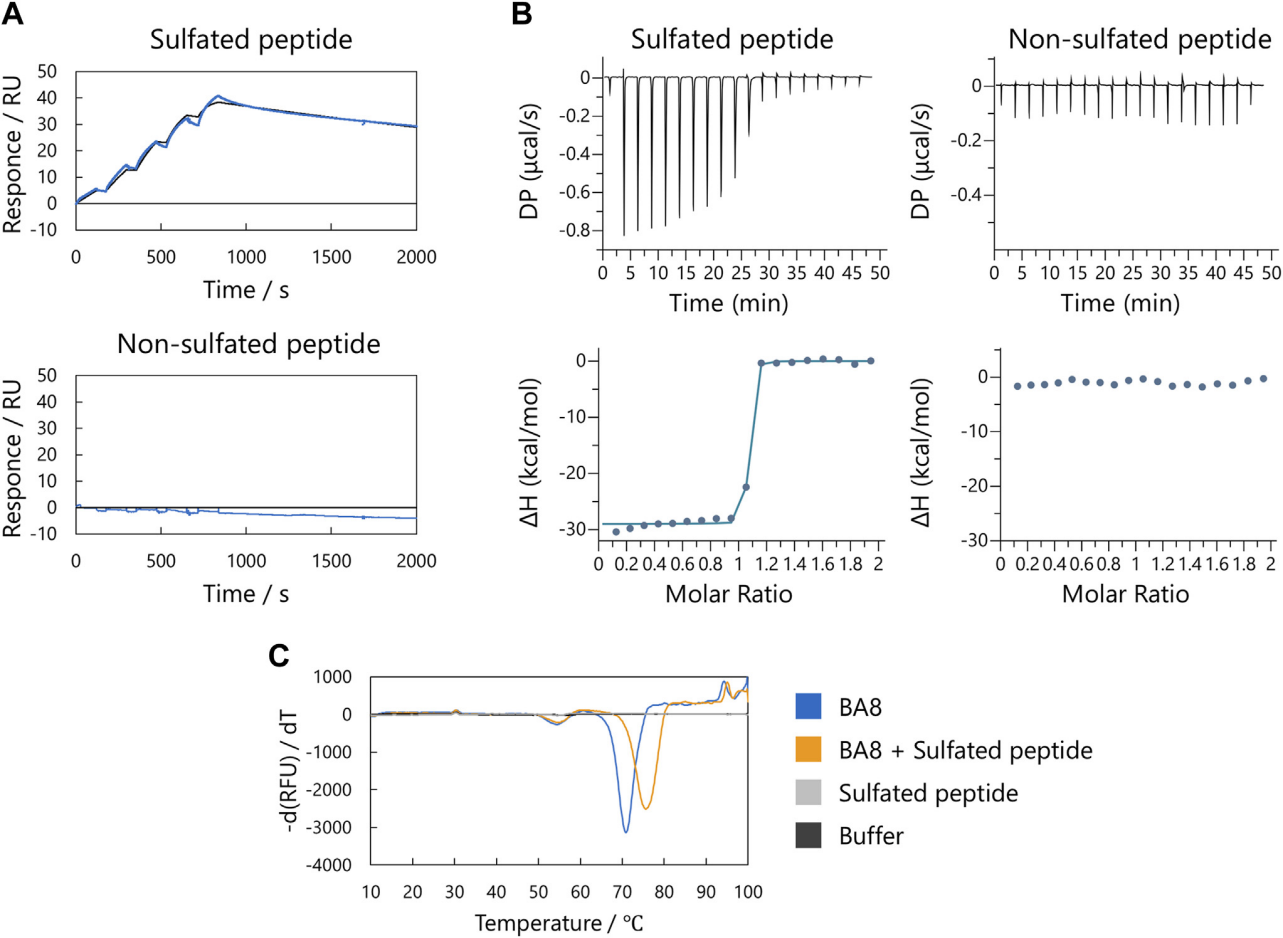
**Molecular interaction analysis of Fab and peptides.***A* and *B*, binding of BA8 to sulfated and non-sulfated peptides. Both (*A*) SPR and (*B*) ITC results suggested that BA8 specifically bound to the sulfated peptide. In the SPR assay, peptides at concentrations ranging from 2 to 162 nM were used as the analyte. *C*, DSF measurement of BA8. After forming a complex with the sulfated peptide in the ITC measurement, BA8 showed a *T*_m_ that was 4.8 °C higher than that of BA8 alone.

To quantitatively evaluate the intermolecular interaction between BA8 and the sulfated N-terminal peptide of CCR5, we prepared a Fab fragment of BA8 as a recombinant protein (Fig. S2, *A* and *B*) and conducted kinetic interaction analysis using SPR. The Fab was immobilized on a sensor chip using amine coupling, and the synthesized sulfated and non-sulfated N-terminal peptides of CCR5 were used as the analytes. The sulfated tyrosine peptide exhibited high binding affinity in the nanomolar range, while the non-sulfated peptide showed no binding within the tested concentration range (Fig. 2*A*, Table 1).

**Table 1:** The kinetic parameters for the BA8-sulfated peptide interaction in SPR analysis

Name	*k*_on_ ($mol\cdot L^{-1}\cdot s^{-1}$)	*k*_off_ ($s^{-1}$)	*K*_D_ ($mol\cdot L^{-1}$)
Wild Type	2.51 × 10^5^	2.03 × 10^−4^	8.09 × 10^−10^
S30 A	2.27 × 10^5^	3.27 × 10^−4^	1.44 × 10^−9^
Y31 A	6.75 × 10^5^	1.25 × 10^−2^	1.86 × 10^−8^
S32 A	2.55 × 10^5^	4.94 × 10^−4^	1.94 × 10^−9^
R34 A			
W46 A	1.82 × 10^5^	3.98 × 10^−3^	2.18 × 10^−8^
S52 A			
N55 A	2.19 × 10^5^	3.95 × 10^−4^	1.80 × 10^−9^
Y57 A	1.34 × 10^5^	5.98 × 10^−2^	4.46 × 10^−7^
Y57 F	2.98 × 10^5^	7.03 × 10^−4^	2.36 × 10^−9^
LC S98 A	1.94 × 10^5^	4.67 × 10^−4^	2.41 × 10^−9^

The association rate constant (*k*_on_, *k*_a_), dissociation rate constant (*k*_off_, *k*_d_), and dissociation constant (*K*_D_) between BA8 mutants and the sulfated peptide were calculated from the SPR analysis.

The bindings of R34 A and S52 A mutants to the peptide were too weak to analyze the proper kinetics parameters.

To determine the thermodynamic parameters accompanying the interaction, we performed an ITC analysis (Fig. 2*B*, Table 2). Consistent with the SPR results, the sulfated tyrosine peptides showed exothermic binding to the Fab, and the binding affinity was so high that it could not be accurately measured under the ITC conditions used. Conversely, the non-sulfated peptides did not exhibit any exothermal reaction, suggesting that it did not bind to BA8.

**Table 2:** The thermodynamic parameters for the BA8-sulfated peptide interaction in ITC analysis

Name	N (sites)	*K*_D_ ($mol\cdot L^{-1}$)	Δ*H* (kcal/mol)	Δ*G* (kcal/mol)	−*T*Δ*S* (kcal/mol)
Wild Type	0.826		−28.7		
S30 A	0.892		−25.8		
Y31 A	0.839	4.41 × 10^−9^	−22.8	−11.4	11.4
S32 A	0.903		−26.0		
R34 A					
W46 A	0.824	6.10 × 10^−9^	−27.2	−11.2	16.0
S52 A	0.796	7.26 × 10^−7^	−16.6	−8.38	8.15
N55 A	0.844		−27.6		
Y57 A	0.854	7.37 × 10^−8^	−21.7	−9.73	12.0
Y57 F	0.896	1.05 × 10^−8^	−25.0	−10.9	14.1
LC S98 A	0.924		−26.2		

The dissociation constant (*K*_D_) and binding enthalpy (ΔH), Gibbs free energy (ΔG), and binding entropy (ΔS) between BA8 mutants and the sulfated peptide were calculated from the ITC analysis. T was the experimental temperature, 297 K.

The binding of R34 A to the peptide was too weak to analyze the thermodynamic parameters.

On the contrary, the bindings of Wild type, S30 A, S32 A, N55 A, and LC S98 A were too strong to analyze the dissociation constant, and the following parameters, ΔG and ΔS.

To assess the effect of sulfated peptide binding on the thermal stability of BA8, we performed differential scanning fluorimetry (DSF) measurements (Fig. 2*C*) (25). The melting temperature (*T*_m_) of BA8 forming a complex with the sulfated peptide was 75.6 °C, whereas the pre-binding *T*_m_ was 70.8 °C. In contrast, we observed no change in *T*_m_ with the addition of non-sulfated peptides, indicating that BA8 is stabilized by binding to the sulfated peptide.

Collectively, these experiments demonstrated that BA8 specifically recognizes the sulfated tyrosines on the N-terminal peptide of CCR5.

### Structural analysis of fab-peptide complex

**Figure 3:**
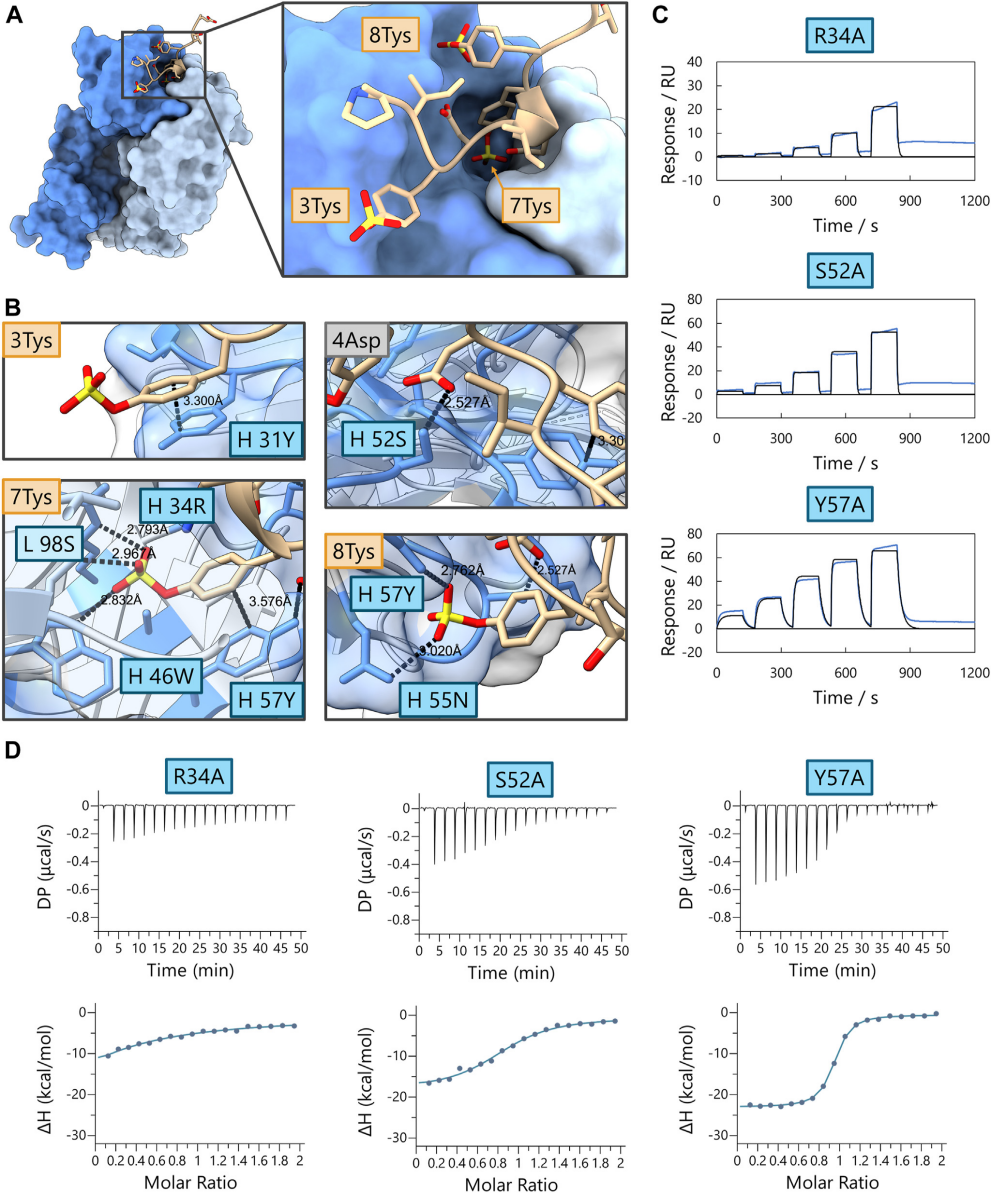
**Crystal structure of the BA8-peptide complex.***A*, overview of the BA8-sulfated peptide complex structure. The heavy chain (HC) of BA8 is shown in *dark blue*, and the light chain (LC) is shown in *light blue*. The close-up view clearly shows that 7Tys is deeply embedded at the variable region of both chains. *B*, hydrogen bonds and π-π interactions between BA8 and the peptide, as suggested by the PDBe PISA Server analysis. Results of (*C*) SPR and (*D*) ITC analyses of three mutants that showed significant loss of binding. In the SPR analysis, the sulfated peptide concentration used as the analyte was 10 times higher (20 nM to 1620 nM) than that in Fig. 2. These results suggest that residues 34R, 52S, and 57Y are hotspot residues for binding to the sulfated peptide.

To elucidate the recognition mechanism of BA8 for sulfated peptides, we conducted an X-ray crystallographic analysis for the BA8-sulfated peptide complex. We determined the crystal structure of the complex at a resolution of 1.8 Å from the crystals derived from the mixed solution of BA8 and the sulfated peptide (Fig. 3*A*, Fig. S3, Table S1, Movie S1). Hereafter, sulfated tyrosines are denoted as Tys followed by their position number. The structure revealed that 7Tys was deeply inserted at the interface between the heavy chain (HC) and light chain (LC), strongly indicating that 7Tys is essential for peptide recognition by BA8. The sulfate group of 8Tys was also present at the interface, suggesting its contribution to the interaction. Conversely, the phenyl group of 3Tys was at the interface with BA8, but the sulfate group was oriented in the opposite direction, suggesting that its sulfation may not contribute to the binding.

To further dissect how molecular recognition occurs, we evaluated the contribution of individual residues of BA8 to the recognition of the sulfated peptide. We analyzed the crystal structure using the PDB PISA server, and we conducted mutation analyses in which the residues predicted to form hydrogen bonds or located at the interface were mutated to alanine (Fig. 3*B*). Because the heavy chain's Y57 is suggested to contribute to hydrogen bonding with the sulfate group of 8Tys and the π-π interaction with the phenyl group of 7Tys in the PDBe PISA server, we prepared both alanine and phenylalanine mutants. The binding affinities of these mutants for the sulfated peptide were measured using SPR and ITC (Fig. 3, *C* and *D*, Tables 1 and 2).

The R34 A mutant exhibited the most significant change in binding affinity, highlighting its critical role in the recognition of 7Tys, as suggested by hydrogen bonding in the crystal structure. The S52 A and Y57 A mutants also showed substantial changes in binding affinity, classifying them as hotspot residues (26). The crystal structure suggested that S52 interacts with 4Asp of the peptide, indicating its importance in maintaining peptide interaction regardless of sulfation. Given that BA8 did not bind non-sulfated peptides, S52 likely supports other residues to achieve the specific binding to the sulfated peptide. The Y57 A mutant exhibited significantly reduced affinity, while the reduced affinity of the Y57 F mutant was limited. This result suggested that the π-π interaction with the phenyl group of 7Tys was more influential than the interaction with the sulfate group of 8Tys (Figs. S4 and S5). Mutants Y31 A and W46 A also showed reduced affinities, which indicated the importance of the π-π interaction of Y31 with 3Tys and that of the hydrogen bond of W46 with the sulfate group of 7Tys in the recognition of the sulfated peptide (Figs. S4 and S5). In contrast, mutations S30 A, S32 A, and N55 A of the heavy chain and S98 A of the LC did not affect the binding affinity for the sulfated peptide (Figs. S4 and S5).

We evaluated the stabilizing effect of peptide binding on each mutant using DSF (Fig. S6). The results revealed a high correlation between the changes in *T*_m_ with binding affinities determined from SPR measurements. This suggests that interactions between hotspot residues and the sulfated peptide contribute to the stabilization of the antibody.

To understand how each residue in BA8 contributes to peptide binding from a viewpoint of dynamics, we conducted MD simulations of antibody residues in both the free state and in complex with the peptide. We conducted the simulations, which were initiated using the crystal structure as the starting structure for the complex, using the Charmm36 m force field and GROMACS2022 software (27, 28). For the unbound antibody, the peptide was removed from the crystal structure, whereas the sulfate group was deleted from the crystal structure for the non-sulfated peptide complex.

Superimposition of the overall structure of the complex of BA8-sulfated peptide and BA8-non-sulfated peptide, and that of the unbound antibody revealed no significant differences in the root mean square deviation (RMSD) of the antibody during the MD simulation (Fig. S7*A*). The root mean square fluctuation (RMSF) of the Cα atoms of each residue also did not differ significantly between the two states (Fig. S7*B*). However, for the amino acid residues we previously identified as being crucial for binding, the RMSF of atoms constituting side chains differed notably among the complexes and the free antibody (Fig. S7*C*). For example, the side chains of R34 and Y57 displayed restricted movement due to interactions with the sulfated peptide, and similar tendencies were observed for W46. These observations suggested that the binding of the Tys stabilized the specific side chain conformations of certain key residues, thereby contributing to the high specificity and affinity of the BA8 antibody for the sulfated CCR5 N-terminal peptide. The MD run movie showed that the non-sulfated peptide, which did not exhibit binding in SPR and ITC, did not dissociate from the antibody in the MD simulations, possibly because MD simulations are highly influenced by the initial structure and can sometimes be trapped in an artificially stabilized local minimum due to an unnatural starting model (29, 30). In conclusion, the MD simulations provided additional insight into the contribution of hot spot residues to binding as revealed by the mutation analysis.

### Importance of each sulfation on tyrosine

Shaik *et al.* (16) previously described the complex structure of CCR5 expressed by mammalian cells and the HIV glycoprotein gp120 and reported that the N-terminal tyrosines of CCR5 were partially sulfated, suggesting that not all of the tyrosine residues are necessarily sulfated together (16). Although we demonstrated that BA8 binds to fully sulfated CCR5 N-terminal peptides with high affinity and specificity, we still needed to determine the contribution of each sulfate group on CCR5 to this binding. Therefore, we designed several partially sulfated N-terminal peptides and evaluated their interaction parameters with BA8.

**Figure 4:**
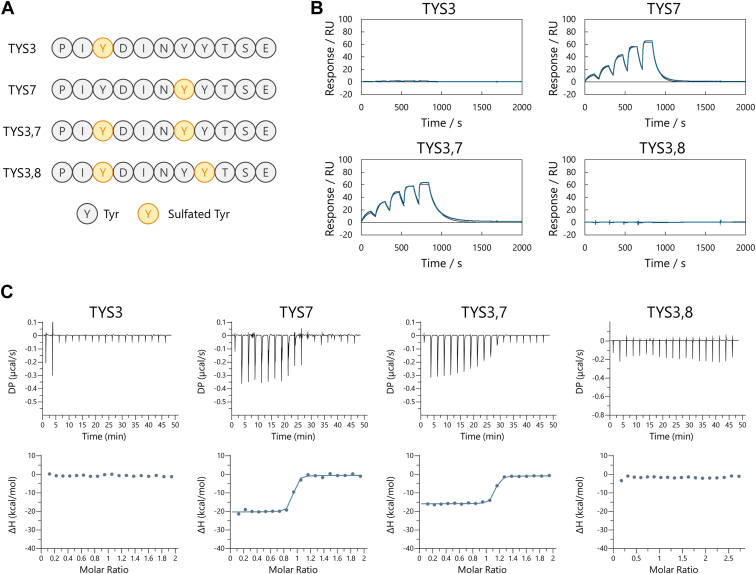
**Interaction analysis with partially sulfated tyrosine.***A*, design of partially sulfated peptides. In addition to the fully sulfated peptide (TYS378) and the non-sulfated peptide (TYR), four other peptides shown in the figure were prepared. *B*, SPR and (*C*) ITC measurements of BA8 with partially sulfated peptides. BA8 bound to TYS7 and TYS37 peptides, which have sulfated Tyr7, but showed no binding to TYS3 and TYS38, which lack this sulfation.

We designed four peptides for this experiment. Peptide one contained 3Tys (TYS3), whose sulfate group forms hydrogen bonds in the interaction with gp120. Peptide two contained 7Tys (TYS7); structural analysis of the BA8 complex suggested that this residue is crucial for binding. Peptide three contained 3Tys and 7Tys (TYS3,7), both of which are sulfated in the CCR5-gp120 complex. Finally, peptide four contained 3Tys and 8Tys (TYS3,8). This peptide lacked the sulfate group at 7Tys, which is likely to be critical for interaction, which allowed us to assess its impact (Fig. 4*A*).

We prepared these partially sulfated peptides and analyzed their interactions with BA8 using SPR and ITC (Fig. 4, *B* and *C*, Tables 3 and 4). TYS3 and TYS3,8 did not exhibit binding, whereas TYS7 and TYS3,7 showed weak binding. These findings indicate that sulfation at 7Tys is essential for BA8 recognition. The absence of sulfation at 8Tys resulted in a roughly 100-fold decrease in affinity, and the enthalpy of binding determined by ITC decreased from −28 kcal/mol to −15 kcal/mol. This result indicates that the hydrogen bond between BA8, specifically Y57, and the sulfate group of Tys8 is critical for maintaining strong binding. Furthermore, the similar affinities of TYS7 and TYS3,7 suggest that the sulfate group at 3Tys is not necessary for peptide recognition by BA8. Thus, the high specificity and affinity of BA8 for the sulfated CCR5 N-terminal peptide likely rely heavily on the sulfation at 7Tys and 8Tys. Collectively, these results indicate that 7Tys is indispensable for recognition and that 8Tys significantly enhances binding strength through additional hydrogen bonding. In other words, BA8 recognizes not only sulfation of the N-terminal peptide of CCR5, but it is also selective about the sulfation site. Therefore, the position or number of sulfate groups significantly affected whether or not BA8 binds as well as its affinity.

**Table 3:** The kinetic parameters for the BA8-partially sulfated peptide interaction in SPR analysis

Name	*k*_on_ ($mol\cdot L^{-1}\cdot s^{-1}$)	*k*_off_ ($s^{-1}$)	*K*_D_ ($mol\cdot L^{-1}$)
TYS3			
TYS7	2.28 × 10^5^	1.54 × 10^−2^	6.78 × 10^−8^
TYS3,7	2.65 × 10^5^	1.19 × 10^−2^	4.50 × 10^−8^
TYS3,8			

TYS3 and TYS3,8 peptides did not show any binding with BA8.

**Table 4:** The thermodynamic parameters for the BA8-partially sulfated peptide interaction in ITC analysis

Name	N (sites)	*K*_D_ (M)	ΔH (kcal/mol)	ΔG (kcal/mol)	−TΔS (kcal/mol)
TYS3					
TYS7	1.17	1.34 × 10^-8^	−14.9	−10.7	4.21
TYS3,7	0.914	7.65 × 10^-9^	−15.8	−11.1	4.68
TYS3,8					

TYS3 and TYS3,8 peptides did not show any binding with BA8.

The stabilization effect of the addition of these partially sulfated peptides on BA8 was evaluated using DSF measurements (Fig. S8). Consistent with the SPR and ITC results, the stabilizing effects of TYS7 and TYS3,7 were lower than those of TYS3,7,8, while TYS3 and TYS3,8 did not change the thermal stability of BA8 at all. Overall, the binding affinity of the peptide and the stabilizing effect were well correlated, indicating that BA8 likely adopts a more rigid structure upon binding to the sulfated peptide, thereby achieving tight binding.

### Recognition of the antigen in the context of the full-length protein

**Figure 5:**
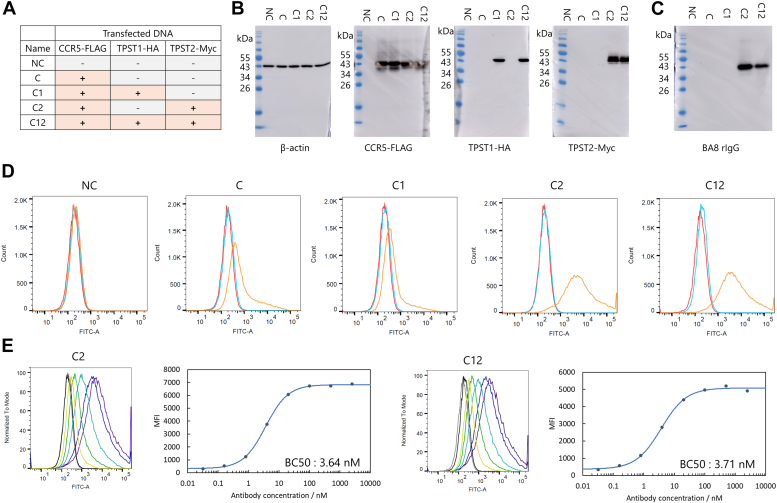
**Biological analysis of the detection of sulfation on CCR5.***A*, Prepared HEK293 cells were transfected with three types of pCAGG vectors to transiently express CCR5, TPST-1, and TPST-2, each conjugated with FLAG tag, HA tag, and Myc tag, respectively. Five different cell types were prepared. *B*, Western blotting using cell lysates. The success of the transfection was confirmed by detecting the tags conjugated to each protein. Detection of β-actin was also performed as a control. *C*, detection of sulfated tyrosine using BA8 as the detection antibody. A CCR5 band was observed only in the cell lysates of C2 and C12, which co-expressed CCR5 and TPST2, suggesting that BA8 specifically detects CCR5 sulfated by TPST2. *D* and *E*, flow cytometry using HEK293 cells. Consistent with the results of the western blotting, strong binding was observed only in C2 and C12 cells. However, very weak binding was also observed in C and C1 cells. Concentration dependant binding of BA8 to C2 and C12 cells was also confirmed.

We prepared model cells with transient overexpression to examine whether BA8 can recognize sulfated full-length CCR5. As referred in the Introduction section, previous research has shown that protein tyrosine sulfotransferases (TPSTs) are responsible for tyrosine sulfation (10, 11, 12). Those two isoforms of TPST (TPST1 and TPST2) and CCR5 genes were transiently transfected in the following five combinations to HEK293 cells: Negative Control (NC); CCR5 (C); CCR5 + TPST1 (C1); CCR5 + TPST2 (C2); and CCR5 + TPST1 + TPST2 (C12). Each protein contained the following tag sequences at the C-terminus: CCR5 – FLAG, TPST1 – HA, and TPST2 – Myc. The overexpression of each protein was confirmed by western blotting using tag antibodies (Fig. 5, *A* and *B*).

We prepared recombinant BA8 as a full-length IgG form for subsequent cell-based assays (Fig. S2, *C* and *D*). BA8 specifically recognized CCR5 in the lysates of cells C2 and C12, in which both CCR5 and TPST2 were overexpressed. This suggests that BA8 detected only CCR5 sulfated by TPST2 (Fig. 5*C*). The absence of other bands indicates that BA8 did not bind to other proteins sulfated by TPST2, demonstrating its specificity toward the amino acid sequence.

We conducted flow cytometry using these cells to confirm that BA8 can recognize sulfated CCR5 expressed on the cell surface (Fig. 5*D*). Consistent with the Western blotting results, BA8 showed clear binding only to cells C2 and C12. However, a slight binding was also observed for cells C and C1, in which only CCR5 or CCR5 and TPST1 were overexpressed. Given that BA8 showed no binding to non-sulfated peptides, CCR5 would be slightly sulfated by endogenous TPST2 in HEK293 cells.

To quantitatively evaluate the binding ability of BA8 to C2 and C12 cells, we plotted the median fluorescence intensity of antibody binding against BA8 concentration (Fig. 5*E*). An increase in the signal with increasing antibody concentration was confirmed, and the binding activity was quantitatively evaluated by fitting with a sigmoid function. The half-maximal binding concentration (BC50) was 3.64 nM for C2 cells and 3.71 nM for C12 cells, which is of the same order as the binding affinity in the SPR analysis with the sulfated N-terminal peptide. These results suggest that BA8 would bind in a similar manner to the full-length sulfated CCR5 on the cell membrane.

### Neutralization of HIV infection

**Figure 6:**
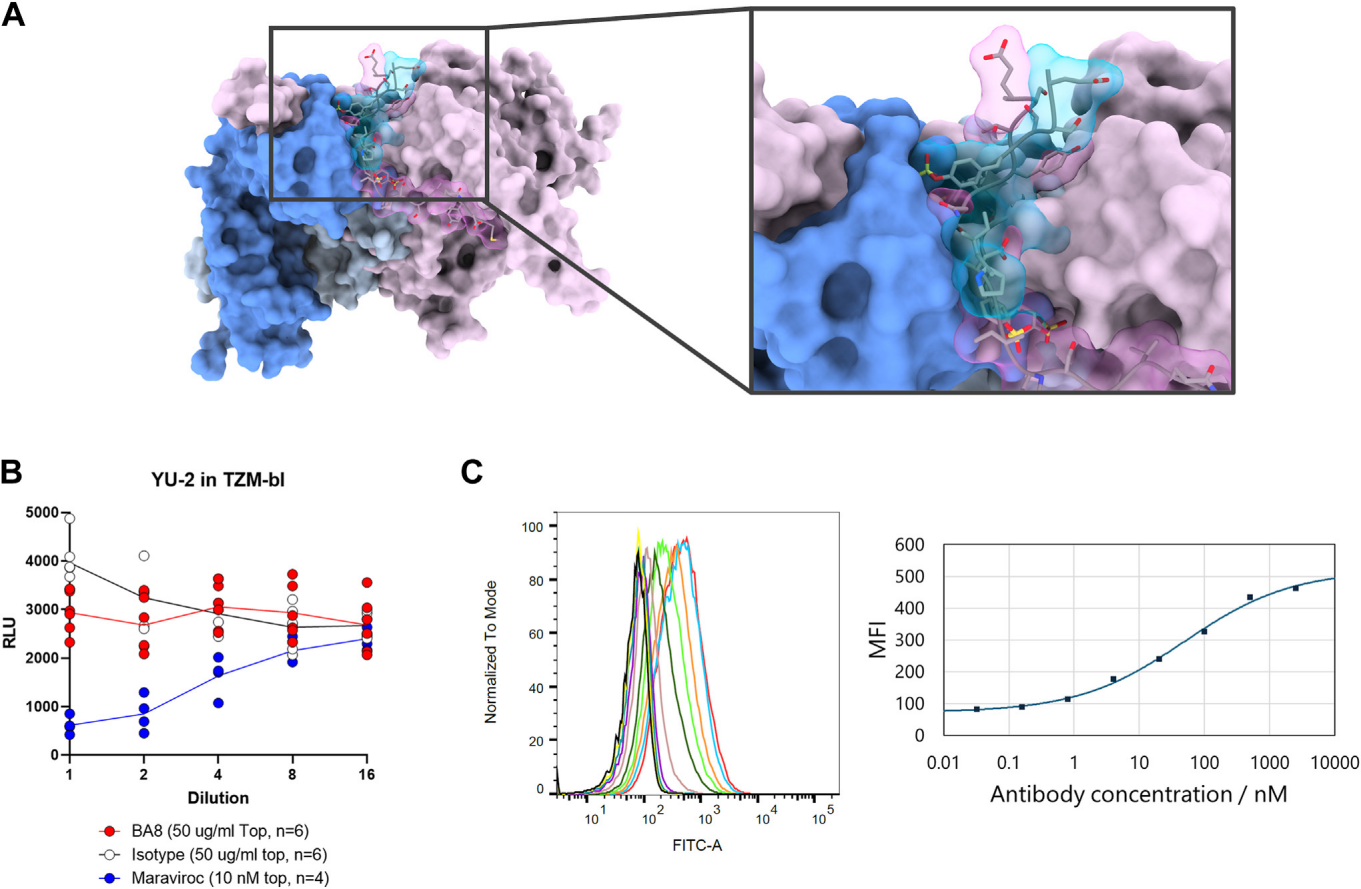
**Neutralizing activity against HIV infection.***A*, superimposition of the BA8-sulfated peptide complex structure with the HIV glycoprotein gp120-CCR5 complex structure. The heavy chain (HC) of BA8 is shown in *dark blue*, the light chain (LC) in *light blue*, and the sulfated peptide bound to BA8 in transparent *blue*. gp120 is shown in *pink*, and the N-terminal peptide of CCR5 bound to gp120 is in transparent *pink*. The most crucial residue for BA8 recognition, 7Tyr, is highlighted in the superimposition. The structure of the peptide bound to BA8 is significantly different from the N-terminal region of CCR5 bound to gp120. *B*, pseudoviral assay using TZM-bl cells. While Maraviroc inhibited HIV infection, BA8 did not significantly inhibit the infection. *C*, flow cytometry using TZM-bl cells. Compared to the binding of BA8 to HEK293 cells overexpressing CCR5 and TPST2, the binding of BA8 to TZM-bl cells was limited, although a concentration-dependent increase in binding was observed.

A comparison of the co-crystal structure of BA8 with the N-terminal peptide and the complex structure of gp120 and CCR5 showed that the peptide structure differs significantly (Fig. 6*A*). Based on this structural analysis, we hypothesized that BA8 binding to the sulfated N-terminal region of CCR5 may inhibit the interaction between gp120 and CCR5, thereby neutralizing HIV infection. Therefore, we attempted to assess the neutralization activity of BA8 on HIV infection.

We first performed flow cytometry using TZM-bl cells to test the binding ability of BA8 to endogenous CCR5 expressed on TZM-bl. We observed a significant binding response, although the binding of BA8 to TZM-bl cells was limited; the BC50 was only 49 nM compared to the higher binding affinity of BA8 to HEK293 cells overexpressing CCR5 and TPST2 (Fig. 6*B*). Subsequently, we conducted a pseudoviral test using TZM-bl cells. The HIV infection inhibitor Maraviroc, which we used as the control, showed concentration-dependent inhibition of viral infection, whereas BA8 did not show inhibitory activity at the maximum concentration of 50 μg/ml (approximately 350 nM) (Fig. 6*C*).

## Discussion

In this study, we generated a sulfated peptide-recognizing antibody (BA8) and elucidated the molecular mechanism responsible for sulfation-specific binding. Previously, we aimed to develop a sulfated peptide-specific antibody and generated an antibody that recognized sulfated peptide. However, that antibody did not directly recognize the sulfate group but rather recognized the dynamic conformation state of the peptide induced by the sulfation (31). In contrast, BA8 recognized the sulfated 7Tys of CCR5, sandwiching it between the HC and LC. Our results also suggested that the sulfation of 8Tys was important for high-affinity binding. Mutational analysis of BA8 suggested that most residues critical for binding are involved in hydrogen bonding with the sulfate group. However, the S52 A mutant of the heavy chain significantly impaired binding to the sulfated peptide despite the lack of interaction with the sulfate group. This Ser52 residue would play a crucial role in the binding of BA8, which is CCR5-specific rather than sulfation-specific. The high affinity and specificity of BA8 toward sulfated N-terminal CCR5 peptides are achieved through the combined interactions of these multiple residues. As in other studies of molecular interactions (32, 33), visualization of the dynamics leading up to the binding of BA8 to the peptide could clarify a more detailed role of each residue, providing a direction for future research.

We demonstrated that the R34 A mutation of the heavy chain of BA8, which diminished the direct binding of Arg34 to sulfated 7Tys *via* hydrogen bond, resulted in significant loss of affinity for the sulfated peptide. On the other hand, DSF measurements revealed another significant characteristic: the *T*_m_ of the unbound wild type was 70.8 °C, while that of the R34 A mutant was higher at 75.0 °C. Upon examining sequence conservation *via* the abYsis server (34), it was found that > 50% of the corresponding residues in rabbit antibodies were Ser, while the Arg presence was > 1%. This suggests that BA8 introduces an unnatural Arg residue into the framework region to recognize sulfated tyrosine, sacrificing thermal stability. Generally, there are two broad molecular design strategies to increase the enthalpy gain from binding: one is to stabilize the post-binding complex, and the other is to destabilize the pre-binding structure (35); this case corresponds to the latter.

Precise characterization of BA8 sulfate recognition at the molecular level allows interpretation of the following biological assay results. In both Western blotting and flow cytometry, BA8 did not show clear binding to CCR5 in cells co-expressing TPST1 and CCR5. As mentioned in the introduction, despite TPST1 and TPST2 having almost the same sulfation mechanism structurally, they are thought to have complementary roles (13). Seibert *et al.* (15) conducted *in vitro* experiments and found that all tyrosines in the N-terminal peptide of CCR5 can be sulfated by either enzyme (15). They also clarified the order in which TPST1 and TPST2 sulfate tyrosine, typically starting with 7Tys or 8Tys, followed by 3Tys, and finally followed by a tyrosine closer to the C-terminus (not included in this study). Additionally, the proportions differ between TPST1 and TPST2; for example, TPST1 often sulfates 7Tys before 8Tys, whereas TPST2 does the opposite, and TPST1 has a minor pathway to sulfate 3Tys or another tyrosine before sulfating 8Tys (15). These findings together with ours suggest three possible reasons why BA8 did not recognize CCR5 under conditions in which TPST1 was solely overexpressed in HEK293 cells: First, TPST1 did not sulfate CCR5; second, TPST1 did not sulfate 7Tys; or third, TPST1 was totally inactive. Testing these possibilities can be performed by proteomic analysis using cell lysates. If the first or second scenario is at work, TPST1 and TPST2 may have a different substrate preference in the cellular environment compared to that observed in the *in vitro* experiment.

Our detailed molecular binding mechanism analysis provided insight into the neutralizing activity of BA8 in the context of HIV infection, as CCR5 is particularly affected by sulfate groups. The limited recognition of CCR5 by BA8 in TZM-bl cells may be due to partial sulfation of CCR5. In an overexpression system using HEK293 cells, the BC50 of BA8 was approximately 3 nM, which corresponds to the binding affinity with peptides when all tyrosines are sulfated. In contrast, the BC50 for binding to CCR5 on the surface of TZM-bl cells was about 50 nM, which corresponds to the binding affinity for peptides when only 7Tyr or both 3Tyr and 7Tyr are sulfated. This result suggests that 8Tyr may not be sulfated in TZM-bl cells.

The circumstantial evidence supporting this hypothesis is related to the fact that BA8 did not exhibit sufficient HIV-neutralizing activity. Liu *et al.* (36) previously investigated the efficiency of HIV infection using chemically sulfated tyrosines on the N-terminus of CCR5 and found that the most critical tyrosine sulfation for infection was at 7Tyr, followed by 3Tyr. Furthermore, those sulfations of 3Tyr and 7Tyr (even without sulfation at 8Tyr) aligned with the sulfation pattern observed in the complex structure of CCR5 and HIV glycoprotein gp120 (16). In other words, while the sulfation of 3Tyr and 7Tyr sufficiently promotes HIV infection, the fact that BA8's site-specific recognition of sulfated tyrosine was not achieved in TZM-bl cells might explain its limited binding to these cells and its insufficient neutralizing activity. We expect that future proteomics analysis will elucidate the tyrosine sulfation status of TZM-bl cells. Nevertheless, BA8, the first antibody that specifically recognizes CCR5 in a sulfation-dependent manner, enables a deeper consideration of the biological activity evaluation. Further, the detailed molecular mechanism of how the antibody specifically recognizes the sulfated CCR5 is expected to lead to the development of antibodies harboring HIV-neutralizing activity.

Moseri *et al.* (37) previously suggested that gp120 may have different CCR5 binding sites depending on the presence of tyrosine, and nuclear magnetic resonance analysis revealed that the residues contributing to the binding differ between sulfated and non-sulfated peptides (37). Although many studies have shown that the sulfation of CCR5 aids HIV infection, gp120 might prepare different binding modes depending on the presence of sulfation. Therefore, achieving high neutralizing activity against HIV with the BA8 antibody alone, which specifically recognizes the sulfate group, may be challenging. Instead, simultaneous use of BA8 with other anti-HIV drugs could be more effective as a treatment for HIV infection. For example, studies have shown that the synthetic N-terminal peptide of CCR5 can inhibit HIV infection (38, 39). Thus, the combined use of a peptide sulfated at Tyr3 and Tyr7 with BA8 might function as an effective HIV infection inhibitor.

Additionally, BA8 recognizes the sulfated peptides using the HC’s complementarity-determining region 1 (CDR1) and CDR2 as well as part of framework region two while utilizing very little of the heavy chain CDR3, which is usually the most diverse and generally the most critical for binding in antibodies (40, 41). This suggests that BA8 has the potential to evolve into an even more useful antibody through further engagement of HCDR3. For example, additional binding to the non-sulfated portions of the peptide *via* CDR3 might enhance the binding affinity to CCR5 independently of the sulfate groups, thereby achieving better HIV infection inhibition. However, this process also could potentially reduce the current high sulfation specificity of BA8, so the design would need to be tailored according to the intended use of BA8.

In summary, we successfully generated an anti-sulfated CCR5 antibody (BA8) and elucidated the molecular mechanism by which it specifically recognizes the sulfated antigen. Although BA8 alone did not inhibit HIV infection of cells, our investigation revealed that BA8 strictly distinguishes the position of the tyrosine sulfate modification and exhibits different affinities. Together with novel antibodies that inhibit HIV infection developed in the future, BA8 can provide insight into the relationship between the tyrosine sulfation state of CCR5 on the cell surface and infection activity. Thus, BA8 should be useful as a research tool and lead to the development of novel therapeutics.

## Experimental procedures

### Peptide synthesis

All peptides used in this study were manufactured by Peptide Institute, Inc. (Osaka, Japan), and correct synthesis was confirmed by mass spectrometry analysis. For immunization and Fab screening, the synthesized peptides were conjugated with KLH.

### Rabbit immunization, library construction, and fab selection

Rabbit antibody was screened as previously described (24). Briefly, New Zealand white rabbits were immunized with KLH-conjugated sulfated peptide. The rabbit that showed the best antibody titer was selected for library construction. Total RNA was extracted from bone marrow and spleen and messenger RNA was purified. cDNA was synthesized and light and heavy chain fragments were amplified and cloned into a Fab-phagemid vector. The library DNA was electroporated into *E. coli* cells and phages were produced by helper phage infection and purified by PEG precipitation. Fabs displayed on phages were selected by biopanning using sulfated peptides. After selection, individual clones were expressed as soluble Fabs and the binding was estimated by ELISA and by flow cytometry using transiently transfected cells expressing CCR5 and TPSTs.

### Preparation of fabs as recombinant proteins

Gene fragments encoding the HC with 6× His added to the C-terminus and LCs from each Fab clone into pcDNA 3.4, an expression vector for mammalian expression systems with an Igκ signal peptide sequence (ThermoFisher Scientific). Expi293 F cells (ThermoFisher Scientific) were co-transfected with expression vectors of HC and LC for each Fab, and the supernatant was collected 5 days after transfection. The supernatant was dialyzed against 20 mM Tris-HCl (pH 8.0), 500 mM NaCl, 5 mM imidazole (binding buffer) and loaded on Ni-NTA resin (QIAGEN) equilibrated with the binding buffer. The resin was washed with 20 mM Tris-HCl (pH 8.0), 500 mM NaCl, 20 mM imidazole (wash buffer), and subsequently the Fabs were eluted by 20 mM Tris-HCl (pH 8.0), 500 mM NaCl, 300 mM imidazole. The eluted Fabs were dialyzed against PBS and further purified by size exclusion chromatography using a HiLoad 26/600 Superdex 200-pg column (Cytiva, Marlborough, MA, USA) equilibrated with PBS. The purity of the purified protein was evaluated by SDS-PAGE. The gels were stained by Coomassie Brilliant Blue R-250. For Western Blotting, proteins were transferred from the gel to nitrocellulose membranes (Cytiva) using a semi-dry transfer system. The membranes were blocked in 5% skim milk in PBS-T (0.05% Tween 20) for 1 h at room temperature. The membranes were incubated with anti-His antibody mAb-horseradish peroxidase (HRP)-DirecT for Fab (MBL Lifescience). Bands were visualized with enhanced chemiluminescence (Cytiva).

### Surface plasmon resonance (SPR)

The bindings of the peptides to Fabs were analyzed using a Biacore 8K instrument (Cytiva). Each Fab was immobilized on the surface of a CM5 sensor chip (Cytiva) by the amine-coupling method according to the manufacturer’s instructions in the condition of pH 5.5. The immobilization level reached to about 3000 Response Unit (RU). PBS containing 0.005% Tween 20 was used as the running buffer. The kinetic data were obtained by injection of increasing concentrations of peptides into the sensor chip. The flow rate of the analyte were set to 30 μl/min. The data were analyzed using BIAevaluation software (Cytiva), and the binding affinity as well as kinetic parameters were calculated by a global fitting of the curves.

### Isothermal titration calorimeter (ITC)

The ITC experiments were performed using a MicroCal PEAQ-ITC (Malvern Panalytical, Malvern, UK). The BA8 Fab fragment antibody, sufficiently dialyzed in PBS, was adjusted to 10 μM for the cells, and the CCR5 N-terminal peptide, dissolved in the dialysate, was adjusted to 100 μM for the syringe. The first injection of 0.5 μl (omitted from the analysis) was followed by 19 injections of 2 μl with 120 s intervals at a constant temperature of 25 °C. The titration syringe was continuously stirred at 750 rpm.

The baselines were smoothly adjusted for the step after each titration saturated and fitted as one set of sites binding in MicroCal PEAQ-ITC Analysis Software (Malvern Panalytical, Malvern, UK).

### Differential scanning fluorometry (DSF)

The DSF thermal stability measurements were performed using a CFX Real-Time PCR System (Bio-Rad). First, 1 μl of 100 × SYPRO Orange stain was diluted with 19 μl of 8.3 μM unbound-BA8 Fab or the solution remaining in the cell after ITC measurement. Next, fluorescence was scanned every minute to obtain melting curves of the solutions as they were heated at 1.0 °C/min. The change in the scanned relative fluorescence unit per unit time was recorded, and the temperature at its peak was considered to be the melting temperature (*T*_m_) (42).

### Crystallization, data collection, and refinement

The well-purified BA8 alone was dialyzed in Tris-HCl 10 mM, NaCl 100 mM and concentrated using an Amicon Ultra MWCO 30000 (Merck Millipore) to approximately 10 mg/ml (approx. 200 μM). Sulfated peptide dissolved in the same buffer was added to the Fab solution to make the concentration 1.2-fold higher than that of Fab. After centrifugation at 20,000 × *g* at 4 °C for 10 min, the supernatant was prepared as the crystallization solution. The initial crystallization screening was carried out using an OryxNano protein crystallization robot (Douglas Instruments). Single crystals for each Fab were obtained in a solution of 10% PEG1000, 10% PEG8000 (solution number seven in Crystal Screen 2, Hampton Research). Suitable crystals were harvested, briefly incubated in mother liquor supplemented with 20% glycerol and 10 mM Tris-HCl (pH 8.0), and transferred to liquid nitrogen for storage until data collection.

Diffraction data from single crystals were collected in beamline BL-5A at the Photon Factory (Tsukuba) under cryogenic conditions (100 K). Diffraction images were initially processed with MOSFLM (43) followed by merging and scaling the data using SCALA of the CCP4 suite (44). The crystal structure was determined following the molecular replacement method using the coordinates of another Fab with the program PHASER (45). The initial model was thoroughly refined using the program REFMAC5 (46) and manually built with COOT (47). Validation was carried out with PROCHECK (48). Data collection and structure refinement statistics are given in the supporting information. The final models were deposited in the Protein Data Bank as PDBID: 9J8A.

### Molecular dynamics simulation

Molecular dynamics (MD) simulations of Fab complexes were performed using GROMACS 2022.3 with the CHARMM36 m force field and the CMAP correction (28). Using the CHARMM-GUI, the Fab structures were solvated with TIP3P water in a rectangular box such that the minimum distance to the edge of the box was 15 Å under periodic boundary conditions. Sodium and chloride ions were added to neutralize the protein charge, then further ions were added to mimic a salt solution concentration of 0.15 M. Each system was energy minimized for 5000 steps and equilibrated with the NVT ensemble (constant Number of particles, Volume, and Temperature) at 298 K for 1 ns. A further production run was performed for 400 ns with the NPT ensemble (constant Number of particles, Pressure, and Temperature), and the time step was set to 2 fs throughout the simulations. A simulation was repeated three times for each system, and the snapshots were saved every 10 ps. UCSF ChimeraX (49) was employed to analyze and visualize the MD trajectories and to render the molecular graphics. To assess the stability of our simulations, we first computed the root mean square deviation (RMSD) of the Cα atoms after superposing the Cα atoms of each complex during the simulations, with reference to the initial structure of the production runs, suggesting that our simulations were well equilibrated after 10 ns. Solvent accessible surface area values as a function of time were calculated using gmx sasa in GROMACS (27).

### IgG preparation as a recombinant protein

IgG expression vectors were constructed by conjugating the heavy chains to the rabbit IgG fragment crystallizable region using an antibody-expressing positive control vector for IgG expression (ThermoFisher Scientific) as a template. Expi293 cells were co-transfected with expression vectors of HC and LC for each IgG, and the supernatant was collected 5 days after transfection. The supernatant was loaded on a rProtein A Sepharose Fast Flow resin (Cytiva) equilibrated with PBS. The resin was washed with PBS, and subsequently, the IgGs were eluted using an IgG Elution Buffer (ThermoFisher Scientific). Next, 2M Tris-HCl (pH 8.0) was quickly added to neutralize the eluted fractions, as the final concentration of Tris is 200 mM. Further purification was conducted by size exclusion chromatography using a HiLoad 26/600 Superdex 200-pg column (Cytiva) equilibrated with PBS. The purity of the purified protein was evaluated by SDS-PAGE. The gels were stained by Coomassie Brilliant Blue R-250 (Wako). For Western Blotting, proteins were transferred from the gel to nitrocellulose membranes (Cytiva) using a semi-dry transfer system. The membranes were blocked in 5% skim milk in PBS-T (0.05% Tween 20) for 1 h at room temperature. The membranes were incubated with HRP-conjugated anti-rabbit IgG (Cell Signaling Technology). Bands were visualized with enhanced chemiluminescence (Cytiva).

### Cell preparation

HEK293 cells stored in liquid nitrogen were initiated into the culture by thawing. After the third passage, at 70% confluency, they were transfected with pCAGGS expression vectors containing CCR5, TPST1, or TPST2 using Lipofectamine 3000 Transfection Reagent (ThermoFisher Scientific). To exclude artifacts caused by the transfection operation itself, empty vectors without the objective protein were transfected if the protein was not to be expressed. After 2 days of culture, the transfected HEK293 cells were gently pipetted off the dish and washed twice with sterile PBS for use in subsequent experiments.

### Western blotting

The cell lysate suspended in RIPA Buffer was placed on ice for 30 min, followed by centrifugation at 20,000 × *g* for 20 min. Complete protease inhibitor was added which was aliquoted to a total protein mass of 20 μg according to the bicinchoninic acid (BCA) assay and then stored frozen. The supernatants of the cell lysates were separated by SDS-PAGE and blotted onto nitrocellulose membranes. Protein bands were detected by incubating the membranes with anti-DDDDK-tag mAb-HRP-DirecT, anti-HA tag mAb-HRP-DirecT, anti-Myc tag mAb-HRP-DirecT (all from MBL Lifescience), or each IgG followed by incubation with horseradish peroxidase (HRP)-conjugated anti-rabbit IgG, HRP-linked antibody (Cell Signaling Technology) at room temperature for 30 min. Bands were visualized with enhanced chemiluminescence (Cytiva). To detect sulfated tyrosine of CCR5, 100 nM BA8 was incubated with the membrane. The composition of the buffer used in all steps was as follows: 20 mM Tris-HCl (pH 7.5), 150 mM NaCl, and 0.01% Tween20.

### Flow cytometry

Cells were aliquoted into 96 Well Conical (V) Bottom Plates (ThermoFisher Scientific) at 50,000 cells per sample and fixed with 2% paraformaldehyde at 4 °C for 30 min. After a PBS wash, 50 μl of goat anti-rabbit IgG Alexa 488 (Invitrogen) diluted 1000-fold by PBS were added to the sample. After incubation at 4 °C for 30 min, PBS washed cells were diluted with PBS containing 2% fetal bovine serum, and flow cytometry was performed using a FACS Canto system (BD Biosciences).

### Pseudoviral assay

The anti-HIV activity of BA8, isotype control antibody, and Maraviroc (Sigma-Aldrich) in a single-round viral infective assay against the YU2 (CCR5 tropic strain) strain in TZM-bl cells. At 48 h post-infection, cells were lysed, and relative light units were measured using a Steady-Glo luciferase assay system and GloMax Discover microplate reader (Promega).

## Data availability

The coordinates and structure factors for the structure of antibodies in complex with sulfated peptide have been deposited in the Protein Data Bank under entry code 9J8A. All other data are available from the authors upon request. Please send request to Kouhei Tsumoto, tsumoto@bioeng.t.u-tokyo.ac.jp.

## Supporting information

This article contains supporting information

## Conflict of interest

The authors declare the following financial interests/personal relationships which may be considered as potential competing interests: The authors used patented technology (WizAmp, US 9,890,414), invented by S. C. J. O. and T. M., for antibody acquisition.

## References

[1] Walsh C.T., Garneau-Tsodikova S., Gatto G.J. (2005). Protein posttranslational modifications: the chemistry of proteome diversifications. Angew. Chem. Int. Ed. Engl..

[2] Cohen P. (1982). The role of protein phosphorylation in neural and hormonal control of cellular activity. Nature.

[3] Choudhary C., Kumar C., Gnad F., Nielsen M.L., Rehman M., Walther T.C. (2009). Lysine acetylation targets protein complexes and Co-regulates major cellular functions. Science (1979).

[4] Greer E.L., Shi Y. (2012). Histone methylation: a dynamic mark in health, disease and inheritance. Nat. Rev. Genet..

[5] Zhou Q., Jaworski J., Zhou Y., Valente D., Cotton J., Honey D. (2020). Engineered Fc-glycosylation switch to eliminate antibody effector function. MAbs.

[6] Arnold J.N., Wormald M.R., Sim R.B., Rudd P.M., Dwek R.A. (2007). The impact of glycosylation on the biological function and structure of human immunoglobulins. Annu. Rev. Immunol..

[7] Stone M.J., Chuang S., Hou X., Shoham M., Zhu J.Z. (2009). Tyrosine sulfation: an increasingly recognised post-translational modification of secreted proteins. N. Biotechnol..

[8] Huttner W.B. (1987). Protein tyrosine sulfation. Trends Biochem. Sci..

[9] Stewart V., Ronald P.C. (2022). Sulfotyrosine residues: interaction specificity determinants for extracellular protein–protein interactions. J. Biol. Chem..

[10] Baeuerle P.A., Huttner W.B. (1987). Tyrosine sulfation is a trans-Golgi-specific protein modification. J. Cell Biol..

[11] Ouyang Y.-B., Moore K.L. (1998). Molecular cloning and expression of human and mouse tyrosylprotein sulfotransferase-2 and a tyrosylprotein sulfotransferase homologue in Caenorhabditis elegans. J. Biol. Chem..

[12] Moore K.L. (2003). The biology and enzymology of protein tyrosine O-sulfation. J. Biol. Chem..

[13] Mishiro E., Sakakibara Y., Liu M.-C., Suiko M. (2006). Differential enzymatic characteristics and tissue-specific expression of human TPST-1 and TPST-2. J. Biochem..

[14] Tanaka S., Nishiyori T., Kojo H., Otsubo R., Tsuruta M., Kurogi K. (2017). Structural basis for the broad substrate specificity of the human tyrosylprotein sulfotransferase-1. Sci. Rep..

[15] Seibert C., Cadene M., Sanfiz A., Chait B.T., Sakmar T.P. (2002). Tyrosine sulfation of CCR5 N-terminal peptide by tyrosylprotein sulfotransferases 1 and 2 follows a discrete pattern and temporal sequence. Proc. Natl. Acad. Sci..

[16] Shaik M.M., Peng H., Lu J., Rits-Volloch S., Xu C., Liao M. (2019). Structural basis of coreceptor recognition by HIV-1 envelope spike. Nature.

[17] Farzan M., Mirzabekov T., Kolchinsky P., Wyatt R., Cayabyab M., Gerard N.P. (1999). Tyrosine sulfation of the amino terminus of CCR5 facilitates HIV-1 entry. Cell.

[18] Scurci I., Akondi K.B., Pinheiro I., Paolini-Bertrand M., Borgeat A., Cerini F. (2021). CCR5 tyrosine sulfation heterogeneity generates cell surface receptor subpopulations with different ligand binding properties. Biochim. Biophys. Acta Gen. Subj..

[19] Blanpain C., Libert F., Vassart G., Parmentier M. (2002). CCR5 and HIV infection. Recept Channels.

[20] Kanmogne G., Woollard S. (2015). Maraviroc: a review of its use in HIV infection and beyond. Drug Des. Devel Ther..

[21] Carter N.J., Keating G.M. (2007). Maraviroc. Drugs.

[22] Ishii M., Nakakido M., Caaveiro J.M.M., Kuroda D., Okumura C.J., Maruyama T. (2021). Structural basis for antigen recognition by methylated lysine–specific antibodies. J. Biol. Chem..

[23] Winter G., Griffiths A.D., Hawkins R.E., Hoogenboom H.R. (1994). Making antibodies by phage display technology. Annu. Rev. Immunol..

[24] Crooks G.E., Hon G., Chandonia J.-M., Brenner S.E. (2004). WebLogo: a sequence logo generator: figure 1. Genome Res..

[25] Huynh K., Partch C.L. (2015). Analysis of protein stability and ligand interactions by thermal shift assay. Curr. Protoc. Protein Sci..

[26] Ran X., Gestwicki J.E. (2018). Inhibitors of protein–protein interactions (PPIs): an analysis of scaffold choices and buried surface area. Curr. Opin. Chem. Biol..

[27] Abraham M.J., Murtola T., Schulz R., Páll S., Smith J.C., Hess B. (2015). GROMACS: high performance molecular simulations through multi-level parallelism from laptops to supercomputers. SoftwareX.

[28] Huang J., MacKerell A.D. (2013). CHARMM36 all-atom additive protein force field: Validation based on comparison to NMR data. J. Comput. Chem..

[29] Bernardi R.C., Melo M.C.R., Schulten K. (2015). Enhanced sampling techniques in molecular dynamics simulations of biological systems. Biochim. Biophys. Acta Gen. Subj..

[30] Hamelberg D., Mongan J., McCammon J.A. (2004). Accelerated molecular dynamics: a promising and efficient simulation method for biomolecules. J. Chem. Phys..

[31] Miyanabe K., Yamashita T., Abe Y., Akiba H., Takamatsu Y., Nakakido M. (2018). Tyrosine sulfation restricts the conformational ensemble of a flexible peptide, strengthening the binding affinity for an antibody. Biochemistry.

[32] Chang C.A., Trylska J., Tozzini V., Andrew McCammon J. (2007). Binding pathways of ligands to HIV-1 protease: coarse-grained and atomistic simulations. Chem. Biol. Drug Des..

[33] Buch I., Giorgino T., De Fabritiis G. (2011). Complete reconstruction of an enzyme-inhibitor binding process by molecular dynamics simulations. Proc. Natl. Acad. Sci. U. S. A..

[34] Swindells M.B., Porter C.T., Couch M., Hurst J., Abhinandan K.R., Nielsen J.H. (2017). abYsis: integrated antibody sequence and structure—management, analysis, and prediction. J. Mol. Biol..

[35] Horn J.R., Kraybill B., Petro E.J., Coales S.J., Morrow J.A., Hamuro Y. (2006). The role of protein dynamics in increasing binding affinity for an engineered Protein−Protein interaction established by H/D exchange mass spectrometry. Biochemistry.

[36] Liu X., Malins L.R., Roche M., Sterjovski J., Duncan R., Garcia M.L. (2014). Site-selective solid-phase synthesis of a CCR5 sulfopeptide library to interrogate HIV binding and entry. ACS Chem. Biol..

[37] Moseri A., Akabayov S.R., Cohen L.S., Naider F., Anglister J. (2022). Multiple binding modes of an N-terminal CCR5-peptide in complex with HIV-1 gp120. FEBS J..

[38] Farzan M., Vasilieva N., Schnitzler C.E., Chung S., Robinson J., Gerard N.P. (2000). A tyrosine-sulfated peptide based on the N terminus of CCR5 interacts with a CD4-enhanced epitope of the HIV-1 gp120 envelope glycoprotein and inhibits HIV-1 entry. J. Biol. Chem..

[39] Dorfman T., Moore M.J., Guth A.C., Choe H., Farzan M. (2006). A tyrosine-sulfated peptide derived from the heavy-chain CDR3 region of an HIV-1-neutralizing antibody binds gp120 and inhibits HIV-1 infection. J. Biol. Chem..

[40] Sela-Culang I., Kunik V., Ofran Y. (2013). The structural basis of antibody-antigen recognition. Front Immunol..

[41] Arnaout R., Lee W., Cahill P., Honan T., Sparrow T., Weiand M. (2011). High-resolution description of antibody heavy-chain repertoires in humans. PLoS One.

[42] Rayaprolu V., Kruse S., Kant R., Movahed N., Brooke D., Bothner B. (2014). Fluorometric estimation of viral thermal stability. Bio. Protoc..

[43] Leslie A.G.W. (2006). The integration of macromolecular diffraction data. Acta Crystallogr. D Biol. Crystallogr..

[44] Winn M.D., Ballard C.C., Cowtan K.D., Dodson E.J., Emsley P., Evans P.R. (2011). Overview of the *CCP* 4 suite and current developments. Acta Crystallogr. D Biol. Crystallogr..

[45] McCoy A.J., Grosse-Kunstleve R.W., Adams P.D., Winn M.D., Storoni L.C., Read R.J. (2007). *Phaser* crystallographic software. J. Appl. Crystallogr..

[46] Murshudov G.N., Vagin A.A., Dodson E.J. (1997). Refinement of macromolecular structures by the maximum-likelihood method. Acta Crystallogr. D Biol. Crystallogr..

[47] Emsley P., Lohkamp B., Scott W.G., Cowtan K. (2010). Features and development of *coot*. Acta Crystallogr. D Biol. Crystallogr..

[48] Koradi R., Billeter M., Wüthrich K. (1996). MOLMOL: a program for display and analysis of macromolecular structures. J. Mol. Graph.

[49] Meng E.C., Goddard T.D., Pettersen E.F., Couch G.S., Pearson Z.J., Morris J.H. (2023). UCSF ChimeraX: tools for structure building and analysis. Protein Sci..

