# Supplementary material for: Specific recognition mechanism of an antibody to sulfated tyrosine and its potential use in biological research
Source: J Biol Chem. 2025 Jan 10;301(2):108176. doi: 10.1016/j.jbc.2025.108176 (PMC11849073; doi:10.1016/j.jbc.2025.108176)
Supplement: Table S1 and Figure S1-S8 [file mmc2.docx]

Supplementary Information

Table S1. Data and refinement statistics of the crystal structure.

| Property | Value | Source |
| --- | --- | --- |
| Space group | P 1 21 1 | Depositor |
| Cell constants a, b, c, α, β, γ | 50.13 Å 70.94 Å 60.98 Å 90.00˚ 97.93˚ 90.00˚ | Depositor |
| Resolution (Å) | 40.68 – 1.75 40.68 – 1.75 | Depositor  EDS |
| % Data completeness | 98.5 (40.68 – 1.75) 98.5 (40.68 – 1.75) | Depositor  EDS |
| R*_merge_* | 0.13 | Depositor |
| R*_sym_* | (Not available) | Depositor |
| < *I / σ (I)* > | 2.10 (at 1.75 Å) | Xtriage |
| Refinement program | REFMAC 5.8.0425 | Depositor |
| R, R*_free_* | 0.165, 0.206 0.175, 0.211 | Depositor DCC |
| R*_free_* test set | 1309 reflections (3.06%) | wwPDB-VP |
| Wilson B-factor (Å^2^) | 15.8 | Xtriage |
| Anisotropy | 0.594 | Xtriage |
| Bulk solvent k*_sol_* (e / Å^3^) | 0.35, 46.9 | EDS |
| L-test for twinning^2^ | < \|L\| > = 0.49, < L^2^> = 0.33 | Xtriage |
| Estimated twinning fraction | No twinning to report | Xtriage |
| F*_o_*, F*_c_* correction | 0.96 | EDS |
| Total number of atoms | 3792 | wwPDB-VP |
| Average B, all atoms (Å^2^) | 19.0 | wwPDB-VP |

The PDB ID is 9J8A. Further information can be found in structure validation report.

Movie S1. The electron density map of the crystal structure.

The movie of the electron density map shown in Figure S3 with rotation.


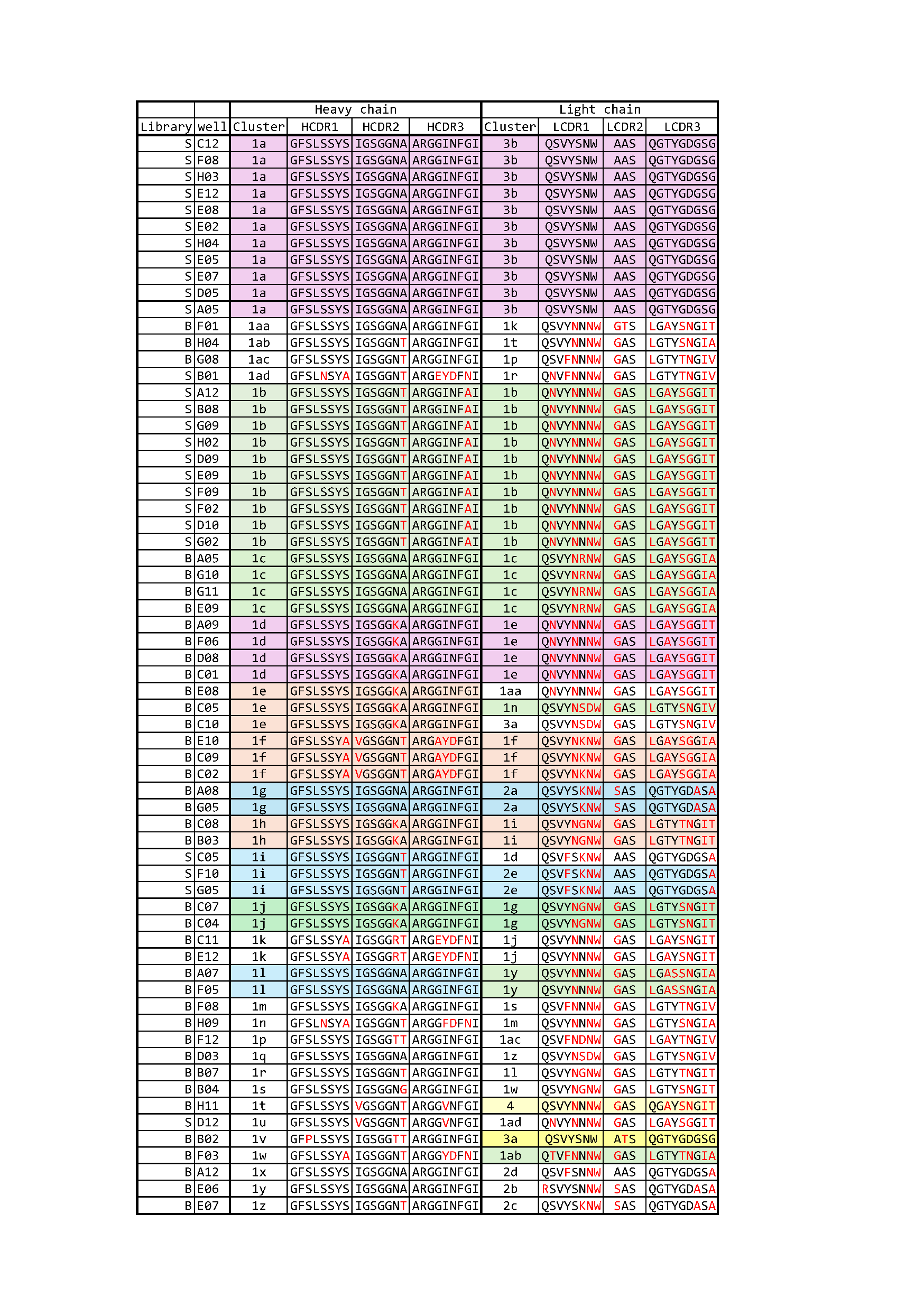


Fig. S1. Sequences of the hit clones from phage display.

CDR sequences of hit clones obtained from two libraries derived from the spleen (S) and bone marrow (B). Residues in red are those that differ from the most common sequences in the HC 1a group and LC 3b group.


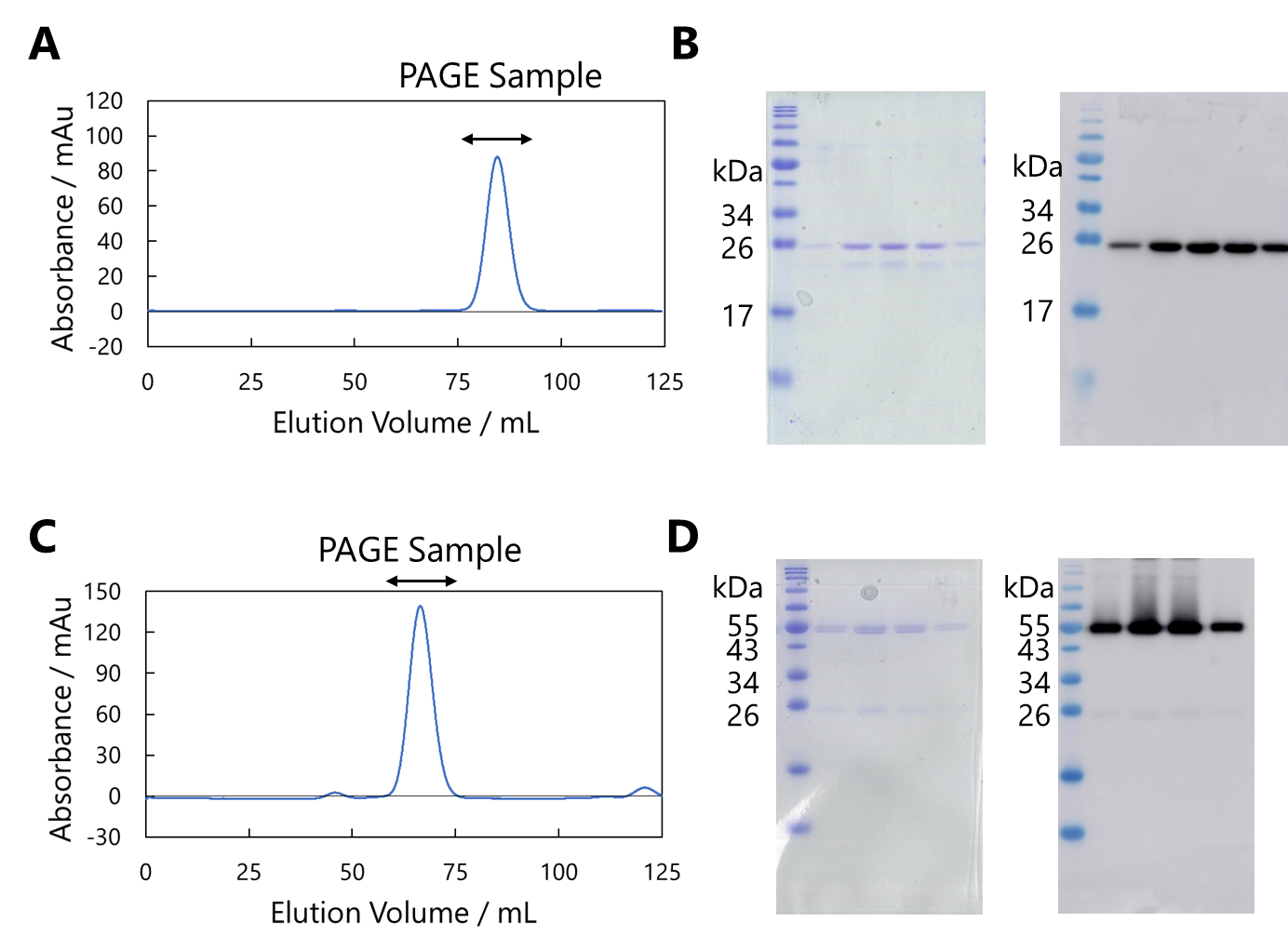


Fig. S2. Preparation of BA8 Fab and IgG.

A) and B). Expression and purification of the BA8 Fab. A) BA8 Fab expressed in Expi293 cells was purified to high purity via IMAC and SEC. B) Sampling of the peak fraction followed by SDS-PAGE confirmed the purity of BA8 Fab, as shown by CBB staining (B left) and western blotting (B right). C) and D). Expression and purification of BA8 IgG. C) BA8 IgG expressed in Expi293 cells was purified to high purity via Protein A column chromatography and SEC. D) Sampling of the peak fraction followed by SDS-PAGE confirmed the purity of BA8 IgG, as shown by CBB staining (D left) and western blotting (D right).


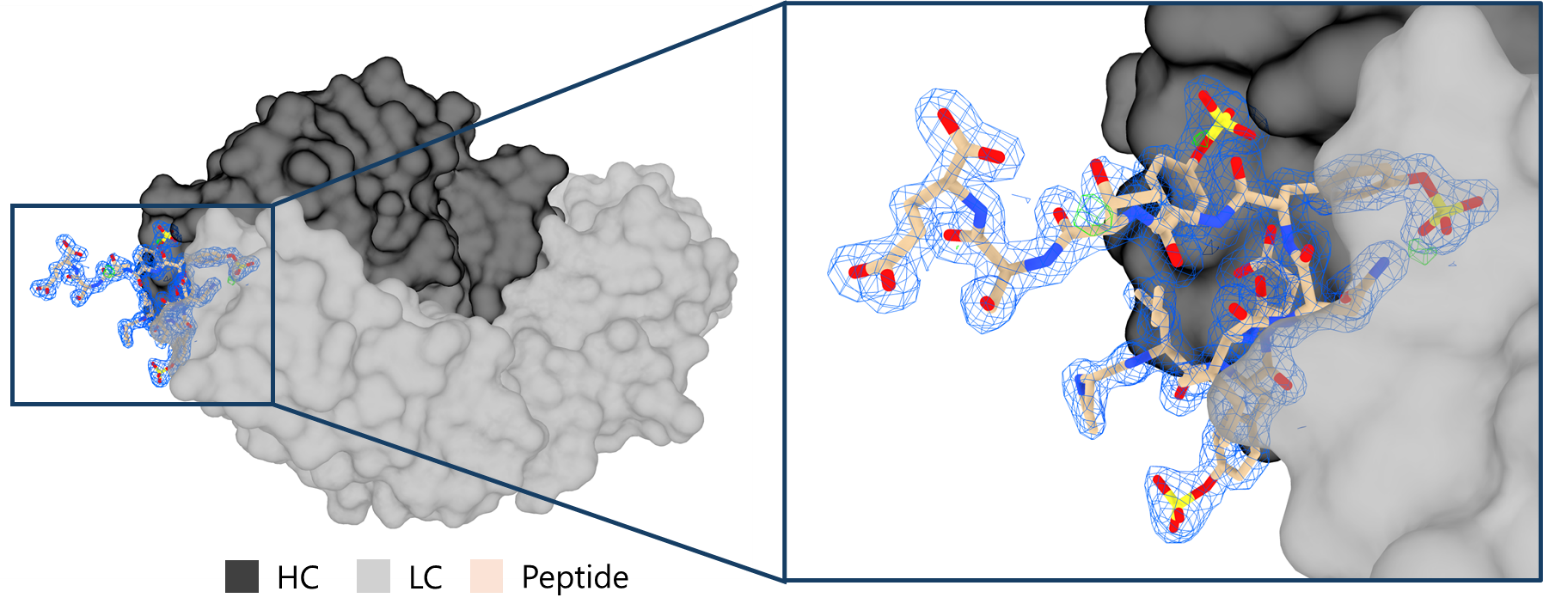


Fig. S3. The electron density map of the crystal structure.

The electron density map displayed both the 2Fo*-Fc** and Fo-Fc maps within 2.0 Å of the peptide. The 2Fo-Fc map was contoured at 1.5σ and shown in blue, representing the overall electron density of the peptide. The Fo-Fc difference map was contoured at ±3.0σ, with positive density shown in green, and negative density in red. The electron density around the peptide was clearly visible and well-defined, indicating that the peptide was accurately modeled within the crystal structure.

*Fo : Observed data, **Fc : Calculated model


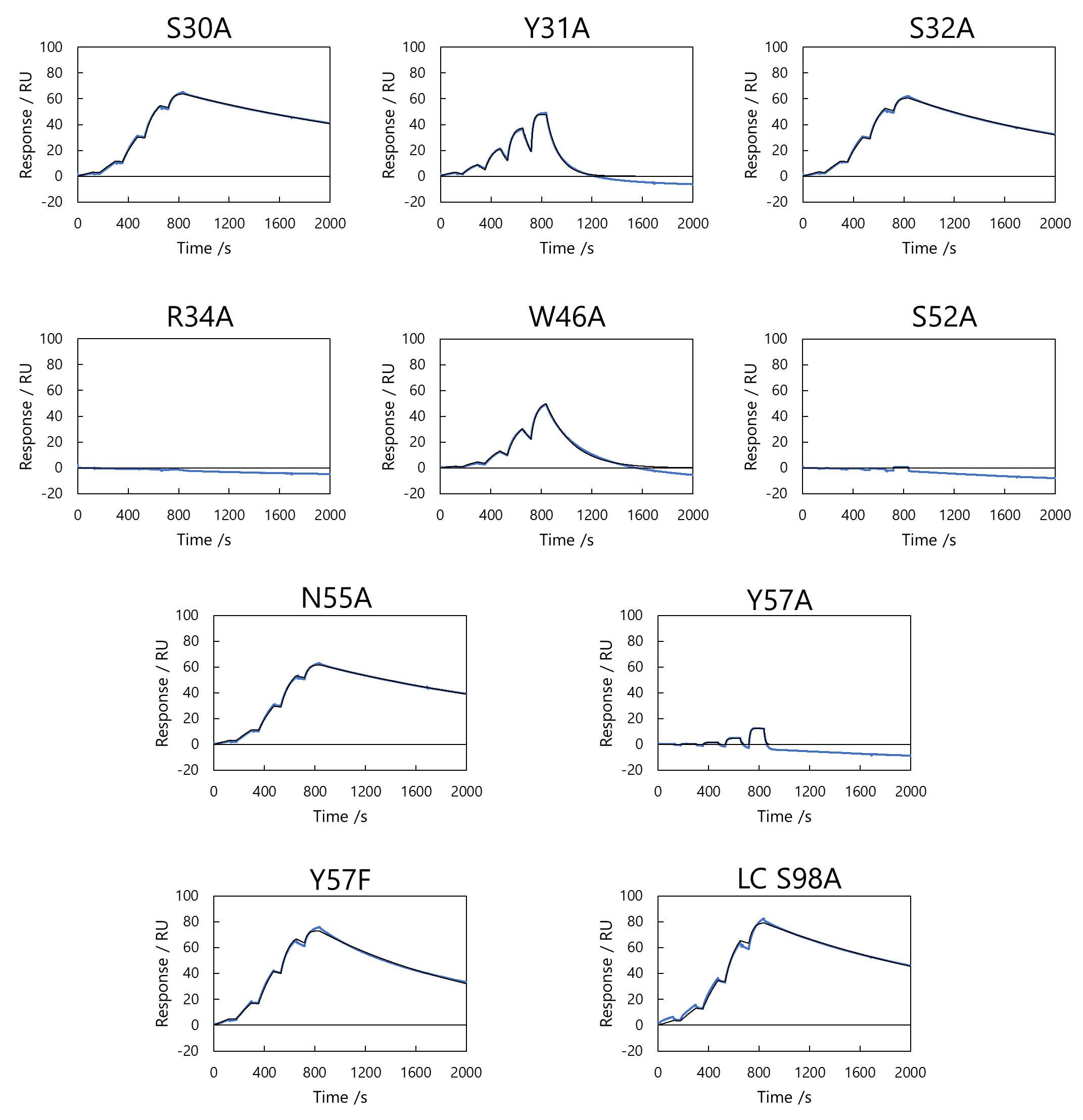


Fig. S4. Interaction analysis between sulfated peptide and BA8 mutants (SPR).

Interaction analysis of BA8 mutants with sulfated peptides was performed using SPR. The analyte consisted of sulfated peptides ranging from 2 to 162 nM. R34A, S52A, and Y57A showed little to no binding, while Y31A, W46A, and Y57F exhibited partial decreases in affinity.


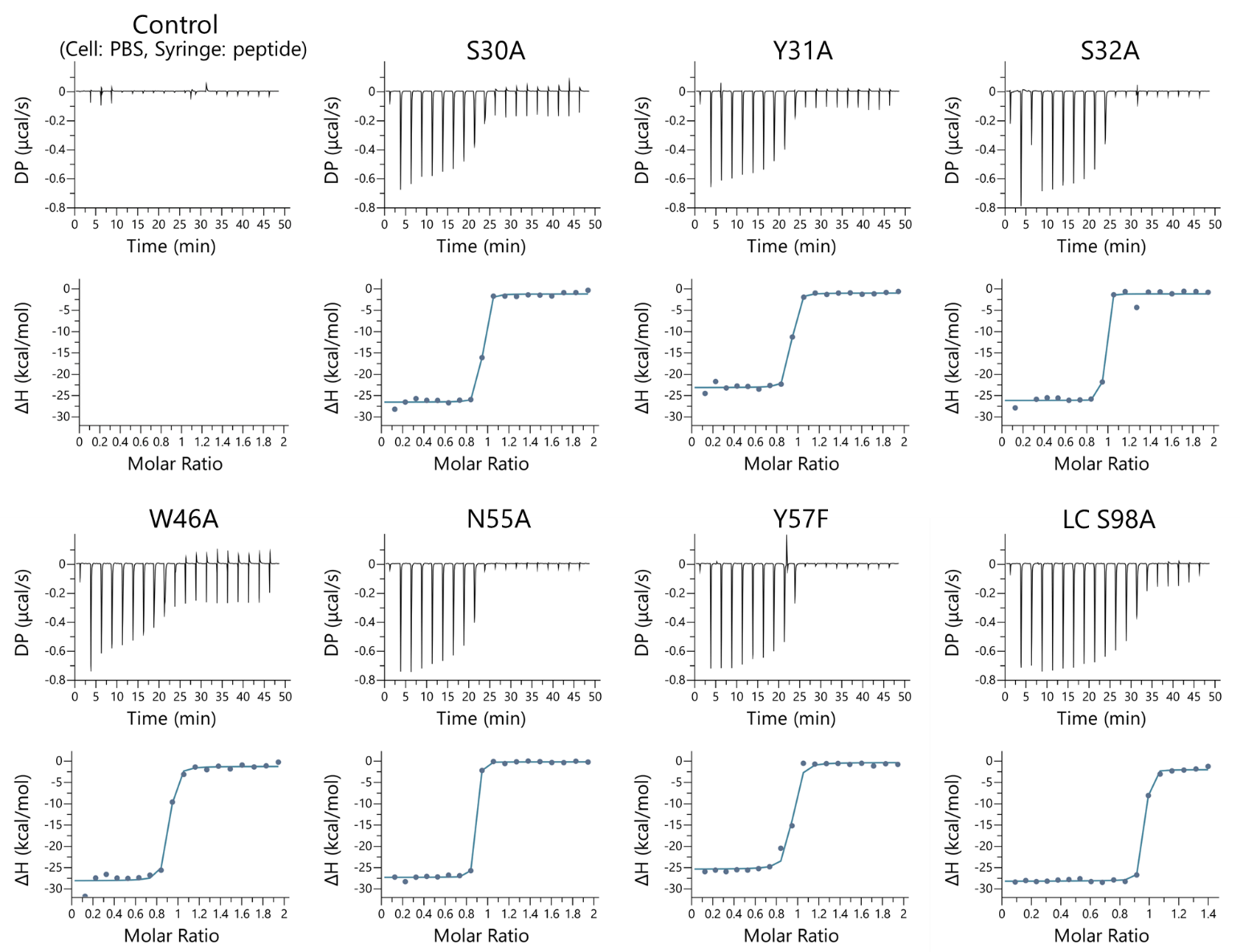


 Fig. S5. Interaction analysis between sulfated peptide and BA8 mutants (ITC).

Interaction analysis of mutants not shown in Figure 3 with sulfated peptides was performed using ITC. Y31A, W46A, and Y57F showed partial decreases in affinity.


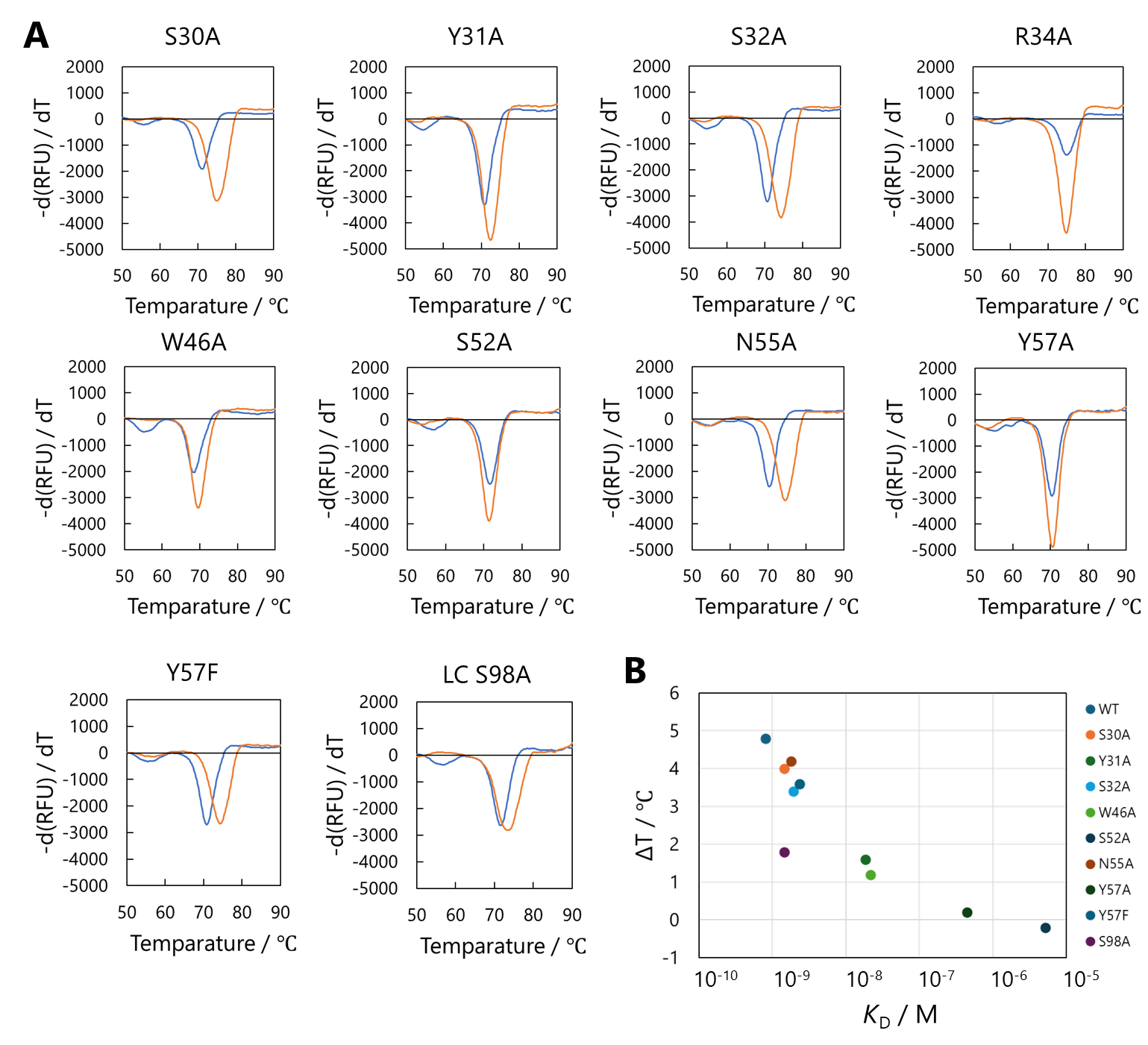


Fig. S6. DSF analysis of BA8 Fab mutants.

A) DSF measurements of each mutant using samples from ITC measurements and the unbound form. Mutants that lacked residues 34R, 52S, and 57Y showed only small changes in *T*_m_, whereas mutants with no change in binding ability, such as S30A, S32A, N55A, and LC S98A, showed *T*_m_ changes comparable to those of the wild type sample. B) Comparison of the *T*_m_ differences of BA8 before and after peptide binding, with the binding affinity of BA8 for the sulfated peptide calculated by SPR. The results suggest that for HC mutants, higher binding affinity corresponds to larger changes in *T*_m_.


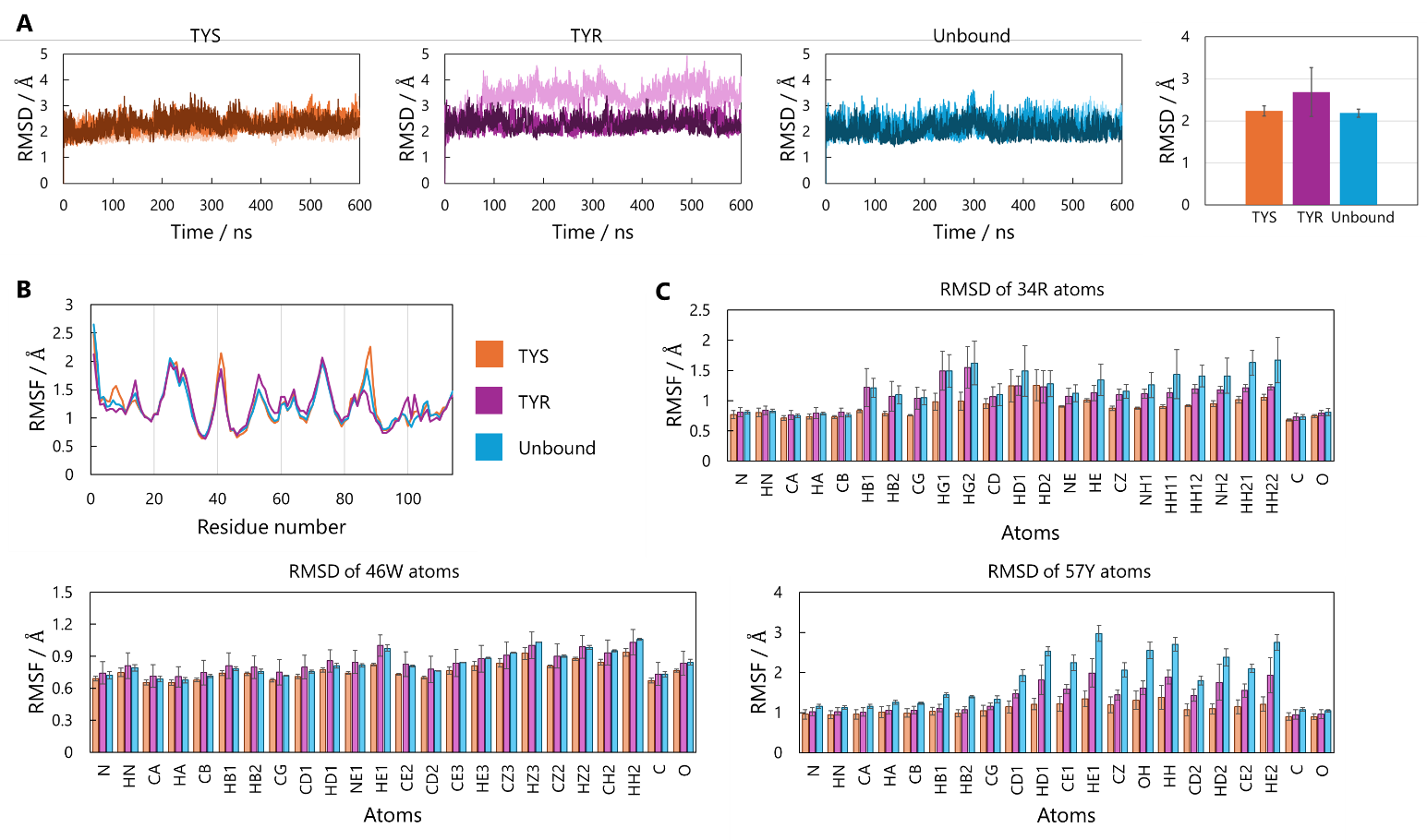


Fig. S7. MD simulation of the BA8 Fab.

MD simulations were performed on three models: the BA8-sulfated peptide complex (TYS) using the crystal structure as the initial structure, the BA8-non-sulfated peptide complex model (TYR) in which the sulfate group was removed from the sulfated tyrosine in the peptide of the crystal structure, and the unbound BA8 model in which the peptide was removed. A) Changes in RMSD values relative to the initial structure during the MD simulation of BA8. The time course and the average RMSD values from 100 ns to 600 ns are shown, but no significant changes were observed. B) Changes in RMSF values relative to the initial structure for the Cα atoms in the VH residues of BA8 during the MD simulation from 100 ns to 600 ns. Similar to the RMSD analysis, no significant differences were observed. C) RMSF analysis of residues 34R, 46W, and 57Y, which were suggested to be important for binding with the sulfate group. While no significant differences were observed in the Cα atoms, the side chain atoms showed an increase in RMSF values in TYR and Unbound compared to TYS, suggesting that the side chains of these residues contribute to complex formation.


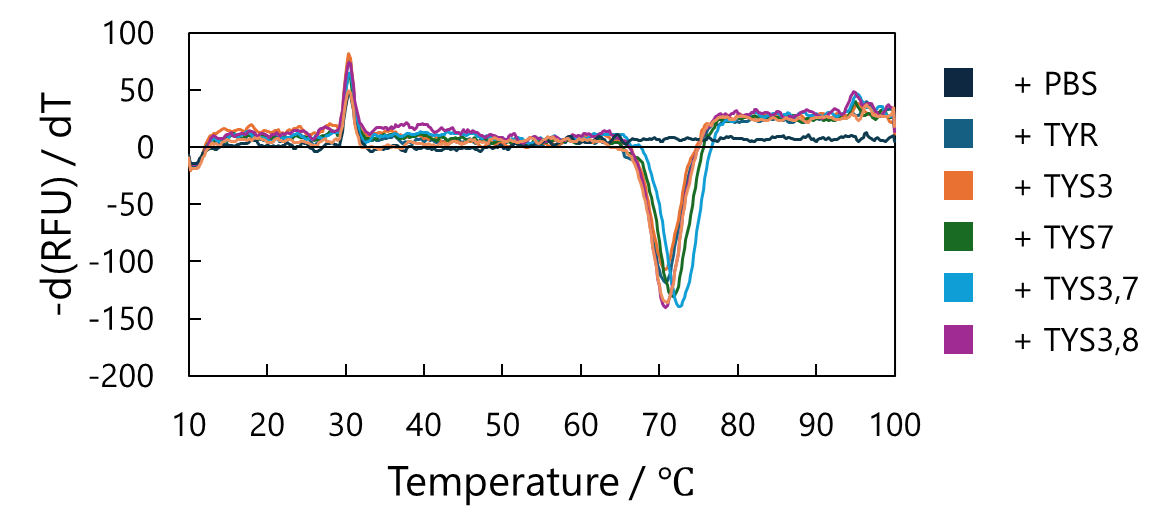


Fig. S8. DSF analysis of BA8 with partially sulfated peptides.

DSF measurements of BA8 Fab before and after binding to partially sulfated peptides suggested that TYS7 and TYS37 contribute to the stabilization of BA8. Similar to Figure S5, changes in *T*_m_s corresponded to the binding affinity with the peptide.
